# Neural correlates of Parkinson’s improvements after combined digital-levodopa therapy: a pilot study

**DOI:** 10.1093/braincomms/fcag148

**Published:** 2026-05-20

**Authors:** Merav Catalogna, Nira Saporta, Tal Tamir, Shira Molcho, Rotem Sivan Hoffmann, Yasmin De Picciotto, Shai Erlich, Sheila Oren, Shahar Shelly, Amir Amedi

**Affiliations:** The Dina Recanati School of Medicine, Reichman University, Herzliya 4610101, Israel; The Baruch Ivcher Institute for Brain, Cognition, and Technology, Baruch Ivcher School of Psychology, Reichman University, Herzliya 4610101, Israel; Remepy Health Ltd, Scientific Department, Ramat Gan 5252247, Israel; Remepy Health Ltd, Scientific Department, Ramat Gan 5252247, Israel; Remepy Health Ltd, Scientific Department, Ramat Gan 5252247, Israel; Radiology Department, Meir Medical Center, Kfar Sava 4428164, Israel; Remepy Health Ltd, Scientific Department, Ramat Gan 5252247, Israel; Remepy Health Ltd, Scientific Department, Ramat Gan 5252247, Israel; Remepy Health Ltd, Scientific Department, Ramat Gan 5252247, Israel; Department of Neurology, Rambam Medical Center, Haifa 3109601, Israel; Rappaport Faculty of Medicine, Technion-Israel Institute of Technology, Haifa 3200003, Israel; The Dina Recanati School of Medicine, Reichman University, Herzliya 4610101, Israel; The Baruch Ivcher Institute for Brain, Cognition, and Technology, Baruch Ivcher School of Psychology, Reichman University, Herzliya 4610101, Israel

**Keywords:** Parkinson’s disease, resting-state fMRI, digital therapy, thalamic nuclei, non-motor symptoms

## Abstract

Parkinson’s disease is characterized by progressive degeneration of dopaminergic neurons within the nigrostriatal pathway, leading to motor and non-motor deficits that become less responsive to chronic dopaminergic pharmacotherapy. Here, we examined whether a multimodal mobile digital intervention, DopApp™, delivering adaptive daily training across sensorimotor, psychological and rehabilitation domains, can induce functional reorganization of thalamocortical motor and limbic networks and augment dopaminergic treatment effects in Parkinson’s disease. To test this hypothesis, we conducted a pilot study in 42 levodopa-treated patients randomized to a 3-week DopApp™ or placebo app intervention. Clinical, psychological and resting-state functional MRI assessments were acquired at baseline and post-treatment. Compared with the placebo group, the active group showed greater motor improvements, as assessed by total scores on the Movement Disorder Society-Unified Parkinson’s Disease Rating Scale (MDS-UPDRS; *P* < 0.001; *d* = 1.22). These improvements were driven by gains in motor function (Part III: *P* < 0.001; *d* = 1.16) and activities of daily living (Part II: *P* < 0.05; *d* = 0.68). Depressive symptoms also improved during the intervention (Beck’s Depression Inventory II: *P* < 0.05; *d* = 0.76), representing a parallel non-motor gain. Seed-based resting-state analyses revealed increased group-by-time connectivity within two parallel thalamocortical pathways. In the motor pathway, connectivity strengthened between the bilateral ventral lateral thalamic nuclei and primary motor cortex (*P*_FDR_ < 0.05), correlating with motor improvement (MDS-UPDRS Part III: *r* = −0.72; *P* < 0.05). In parallel, increased connectivity was observed within the limbic circuit linking the anteroventral thalamus with medial prefrontal, posterior cingulate and hippocampal regions (*P*_FDR_ < 0.05). Connectivity increases within this pathway correlated with improved depressive symptoms (*r* = −0.65; *P* < 0.01) and self-perceived daily function (MDS-UPDRS Part II: *r* = −0.66; *P* < 0.01). Additionally, engagement with specific app modules predicted domain-specific gains, and connectivity changes correlated with these improvements, suggesting a dose–response relationship and parallel circuit-specific neuroplasticity. These findings provide preliminary evidence supporting the integration of scalable digital interventions into precision, drug-digital treatment paradigms for Parkinson’s disease and related neurodegenerative disorders. Moreover, they represent a conceptual advance towards personalized care models that enhance drug efficacy and expand therapeutic impact beyond conventional clinical settings.

## Teaser

Catalogna *et al*. studied how using the smartphone app DopApp™ alongside levodopa, a standard Parkinson’s medication, can impact brain areas related to movement and emotions. They concluded that engagement with the app improved brain functional connectivity and related motor and mood symptoms, supporting the promise of combined digital-drug therapy.

## Graphical Abstract

**Graphical Abstract:**
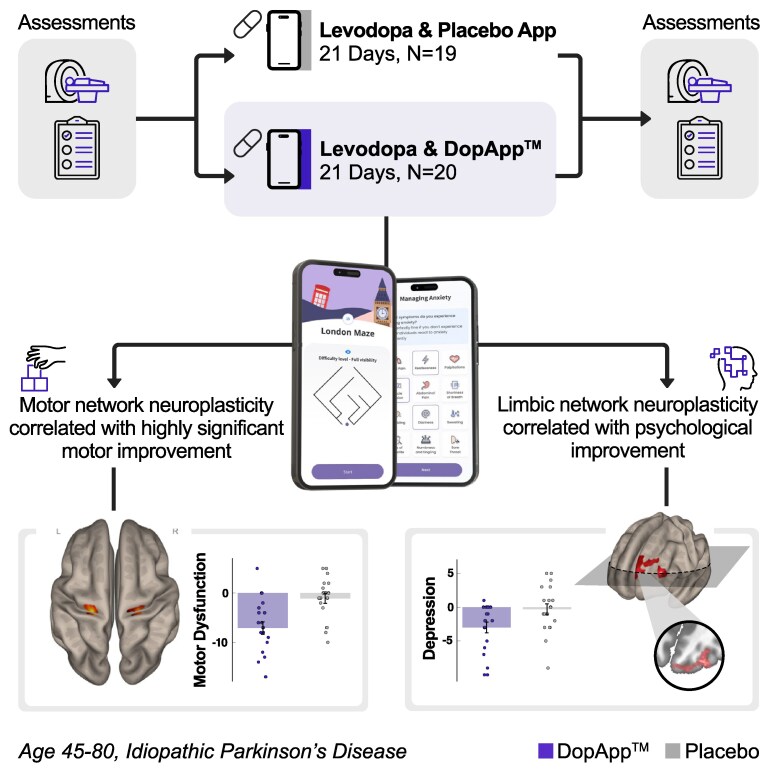

## Introduction

Parkinson’s disease, the second most common neurodegenerative disorder, affects millions worldwide and is a growing global health challenge.^1^ While levodopa remains the cornerstone of treatment, its long-term use is limited by motor complications and its little effect on non-motor symptoms (NMSs),^2,3^ resulting in significant unmet needs in effectively managing both the motor and non-motor aspects of Parkinson’s disease.^4^

Multimodal non-pharmacological therapies, including physical, cognitive and psychological interventions, are recognized as essential to comprehensive Parkinson’s disease management.^5-7^ While multidisciplinary approaches improve both motor and NMSs, access remains limited even in high-income countries.^8,9^ Despite clinical guidelines, the utilization of non-pharmacological interventions remains low due to geographic, financial, logistical and public health educational barriers,^10,11^ a gap that digital health could address, but has yet to efficiently integrate into routine care.^12^ Currently, most Parkinson’s disease digital solutions narrowly focus on motor symptom tracking (e.g. gait and balance) without targeting improvement, and with limited integration of validated psychological or cognitive strategies for NMS.^13,14^ Additionally, very few digital platforms are personalized or have undergone rigorous evaluation of treatment efficacy or underlying mechanisms,^15^ partly due to methodological challenges, such as developing placebo controls for digital therapeutics.^16,17^ Therefore, our first aim was to assess whether digitizing established rehabilitation and psychological strategies improves therapeutic outcomes in levodopa-treated people with Parkinson’s disease (PwP), compared to a placebo intervention, providing a therapist-free and scalable solution.

In addition to exploring therapeutic benefits, this study was guided by a circuit-level mechanistic framework aimed at identifying neural correlates of clinical improvement. Cortico-basal ganglia (BG)–thalamocortical circuits are organized as multiple parallel, functionally specialized yet interacting loops supporting motor, cognitive and limbic functions.^18^ Functional disturbances across these parallel circuits contributes to the core motor and NMSs of Parkinson’s disease.^18-20^ While early resting-state functional MRI (rs-fMRI) studies emphasized cortico-striatal disruptions,^21-23^ the thalamus has been comparatively understudied despite its central role in relaying and integrating input from the BG, cortex and cerebellum.^24,25^ A recent high-resolution rs-fMRI study narrowed this gap, revealing systematically reduced resting-state functional connectivity (rsFC) between sensory and motor thalamic subdivisions and their respective cortical targets in early-stage Parkinson’s disease.^26^ Furthermore, the thalamus’s involvement in both motor^27,28^ and NMSs^29^ highlights its central role in Parkinson’s disease-related circuit pathology and its potential as a target for therapeutic modulation.^30^ Notably, rs-fMRI is sensitive to treatment-induced plasticity: levodopa increases functional connectivity in the ON-state compared to the OFF-state or drug-naïve conditions.^31-33^ Additionally, rehabilitation targeting motor,^34,35^ cognitive or affective functions^36,37^ can further modulate network dynamics.^38^ However, existing longitudinal studies often assess motor and NMSs separately, rarely probing specific functional thalamocortical subcircuits. Guided by this framework, we hypothesized that a multimodal digital intervention engaging multiple functional domains would be associated with distinct rsFC changes within parallel, functionally specific thalamocortical networks, corresponding to improvements in motor and NMSs.

To address the therapeutic gap for scalable and multidisciplinary interventions, we developed DopApp™ (Remepy Inc., New York, NY, USA), a digital therapeutic application designed to augment standard dopaminergic treatment. DopApp™ builds on a previously validated digital protocol that combined a sensorimotor-deprivation task and psychological interventions to promote spatial memory, emotional resilience and immunological regulation in older adults with subjective cognitive decline (SCD), elevated stress and depressive symptoms.^39-41^ The sensorimotor-deprivation component included synchronized visual and auditory stimulation, sensory substitution and deprivation and adaptive gamification via spatial memory tasks. These cognitive neuroscience elements were shown to enhance circuit-level plasticity^42^ and were complemented by psychological strategies targeting emotional regulation. Together, they reduced depressive symptoms and modulated rsFC across cognitive, affective and visuomotor networks, including limbic regions, such as the amygdala and medial temporal lobe (MTL). These same regions are critically implicated in Parkinson’s disease, where structural and functional alterations are consistently linked to NMSs.^43-45^ Adapting this framework, the Parkinson’s disease-specific version also incorporated daily modules targeting additional sensorimotor activations, speech and emotion regulation. These modules were developed with specialists and are aligned with core Parkinson’s disease treatment guidelines.^5-7^ Finally, to optimize digital health interventions, it is essential to determine their dose–response relationship, finding the levels and patterns of engagement yielding the most meaningful benefits.^46^ We therefore examined the relationship between app usage metrics, Parkinson’s disease-scale assessments and imaging outcomes across two core domains: emotion regulation and motor activation. Together, this approach aims to support and extend the therapeutic effect of levodopa by engaging brain circuits that pharmacological treatment alone may not fully activate and to identify links between usage patterns and outcomes.

The therapeutic and neural effects of DopApp™ were evaluated in comparison with a placebo app, guided by three hypotheses: (i) a self-guided, multimodal digital intervention will improve clinical outcomes beyond standard dopaminergic treatment, compared to a placebo app; (ii) app engagement will enhance rsFC within motor thalamocortical circuits, correlating with motor symptom improvement; and (iii) app engagement will enhance rsFC within the limbic thalamocortical and amygdala networks, correlating with affective improvements. By integrating clinical, app engagement and imaging data, this study aims to support the validation of digital therapeutic use in Parkinson’s disease care and related neurodegenerative diseases.

## Materials and methods

### Study design and participants

**Figure 1:**
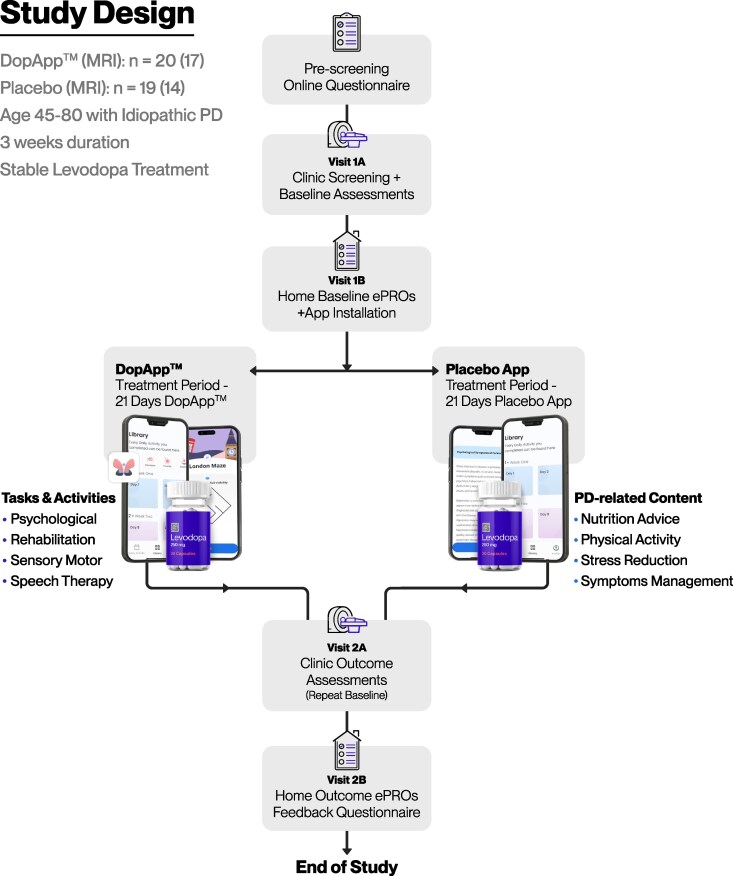
**Study design.** Participants were pre-screened up to 2 months before study initiation via an online questionnaire and a follow-up phone interview with clinical staff. Visit-1A (in-clinic) included informed consent, medical history review, abbreviated physical exam, MDS-UPDRS assessment, cognitive testing [Montreal Cognitive Assessment (MoCA), TMT] and MRI. Eligible participants were then randomized 1:1 to receive either the DopApp™ or placebo app for a 3-week treatment period. The placebo app had a highly similar appearance to the DopApp™ protocol, with identical access and onboarding procedures. Home visit-1B (conducted the day after) involved app installation and completion of PD-specific and psychological questionnaires. Post-intervention visit-2 (in-clinic) repeated the physical exam, Parkinson’s disease-scale and cognitive assessments and MRI. Finally, home visit-2B included Parkinson’s disease-specific, psychological questionnaires alongside a feedback survey. Safety monitoring involved documentation of AEs and reporting of serious adverse events (SAEs) to national regulatory authorities as required

This study was conducted at the Baruch Ivcher Institute for Brain, Cognition & Technology (BCT) within the School of Psychology at Reichman University, Israel, between 11 August 2024 and 23 October 2024 and concluded upon reaching the predetermined recruitment target. Participants were recruited through social media and patient advocacy groups. Further details of the protocol are provided in the Supplementary material. Eligible participants, aged 45–80 years old with a recorded Parkinson’s disease diagnosis and treated with stable daily regimens of levodopa (stable regimen of 150–1500 mg/day for ≥30 days with a maximum of five doses per day), were randomly allocated to either the DopApp™ or placebo app for 3 weeks (Fig. 1). Randomization was performed in a 1:1 ratio with a block size of 4, using a computer-generated randomization table. PwP with additional major unstable neurologic, psychiatric or medical conditions were excluded. Participants taking other Parkinson’s disease drugs were included in the study, and LD equivalence doses were calculated.^47^ All study personnel were blinded to treatment allocation, except for Remepy’s technical staff responsible for app installation and onboarding, and the technical helpdesk when applicable. The study duration was based on previous studies in older adults with SCD,^39,41^ where 2- and 3-week protocols demonstrated behavioural and neuroimaging effects. A similar timeframe was chosen for this Parkinson’s disease study to ensure feasibility and adherence given the population’s characteristics. The study was conducted according to the Declaration of Helsinki guidelines and local institutional regulations. The study was approved by the Reichman University Institutional Review Board (IRB) (P_2024094, on 18 April 2024), and the neuroimaging study protocol was approved by Meir Medical Center’s IRB (122-24-MMC, Israeli registration, MOH_2024-08-06_013567 on 5 August 2024). All participants provided written informed consent prior to their inclusion.

### Treatment

The DopApp™ intervention is a comprehensive multimodal programme integrating cognitive, psychological and rehabilitation-focused exercises across well-established disciplines. It incorporates principles of multisensory (vision, audio) integration, sensory substitution and masking. These techniques were digitized and delivered through short interactive videos, audio and engaging games. DopApp™ interventions were structured into 21 daily routines and included the following components: (i) digital adaptations of relevant fine motor skills, physiotherapy, occupational therapy, speech therapy and dance exercises; (ii) a virtual audio-visual spatial memory navigation exercise based on the principles of sensorimotor integration and gradual visual masking^41,42,48,49^; (iii) psychological interventions adapted from cognitive behavioural therapy (CBT), acceptance and commitment therapy (ACT), mindfulness-based stress reduction (MBSR), mindfulness-based cognitive therapy (MBCT), guided imagery, psychoeducation and attention training; and (iv) daily walking, including at least 15 min of scheduled walking (Supplementary Fig. 1 and Supplementary material).

The app utilized adaptive algorithms to dynamically adjust task difficulty based on real-time performance metrics, engagement and progression. The placebo app had a similar appearance to the DopApp™, and onboarding steps were identical. The placebo intervention protocol consisted of a daily regimen featuring Parkinson’s disease-related content, such as nutritional guidance and the general benefits of physical activity (Fig. 1). All study participants received daily new content, along with app usage reminders, as well as access to an on-demand library of previously completed interventions.

### App data analysis

To evaluate user engagement with the DopApp™ intervention, all app interactions were automatically logged, including timestamps and activity-type identifiers. These data were analysed to quantify usage patterns across the app’s various components and to distinguish between content completed as part of the structured protocol and content accessed voluntarily.

For analysis purposes, app activity was categorized into two main functional domains: (i) Emotion regulation (via media content)—this category included prerecorded video and audio materials such as guided mindfulness sessions, psychoeducational content (the majority of the content) and therapeutic exercise videos in areas such as physical therapy, speech therapy neuro-dance and occupational therapy. (ii) Sensorimotor (via serious sensorimotor games)—activities that integrate sensory input with motor response. All sensorimotor games were interactive tasks requiring active user participation. Users had to engage with the task and meet a minimum performance threshold to progress through the daily protocol. Each game provided immediate feedback, such as scores, star ratings or progression indicators. Games targeted motor, sensorimotor or speech-related functions and included tasks designed to train fine motor skills, vocal control, coordination and spatial processing. Within this category, the sensorimotor-deprivation intervention, a virtual spatial memory navigation exercise, was the most prominent and was therefore also analysed separately.

Content was also classified by use type: (i) Protocol-driven use (required): engagement with daily assigned sessions within the 3-week intervention period. The daily protocol was designed to last ∼30 min; actual engagement times are presented in Supplementary Table 1. (ii) Self-initiated use (extra): any additional, self-initiated engagement with content through the on-demand content library. Specifically, self-initiated games measure, which reflects involvement in cognitively demanding and reward-based tasks, was used as a motivation score. Detailed engagement times are provided in Supplementary Table 1.

Each participant’s usage data was analysed using three summary engagement metrics: (i) Exposure—total time (minutes): the cumulative time spent using a given content type. This reflects overall exposure and adherence to that component. (ii) Daily dose: average daily usage (minutes/day): the mean time spent per day on active days. This captures daily engagement or daily dose for each content type. (iii) Usage consistency—number of days: the total number of calendar days in which a participant engaged with the content. This metric was only calculated for self-initiated use, as protocol-driven use occurred consistently across all 21 intervention days by design. Therefore, usage consistency reflects voluntary long-term engagement and adherence beyond the structured protocol.

### Outcomes and assessments

Participants were assessed at baseline and at the end of study by a blinded rater, from an external medical services centre, with the same clinician conducting both assessments for each participant. The primary therapeutic outcome was the change from baseline in the Movement Disorder Society-Unified Parkinson’s Disease Rating Scale (MDS-UPDRS)^50^ total score (sum of Parts I, II and III) compared with placebo. MDS-UPDRS Part III assessments were conducted in the ON state as determined by the patient’s subjective experience of motor function improvement after taking the medication. Additional outcomes included changes from baseline in MDS-UPDRS sub-parts (Part II, Part III, sum of Parts II and III and item clusters related to speech and fine motor function), the Parkinson’s Disease Sleep Scale (PDSS2),^51^ Parkinson’s Disease Questionnaire 39-item (PDQ-39),^52^ Voice Handicap Index (VHI),^53^ Perceived Stress Scale (PSS-10),^54^ Brief State and Trait Anxiety Inventory (STAIT-5/STAIS-5),^55^ Beck’s Depression Inventory II (BDI-II),^56^ Mental Health Continuum Short Form (MHC-SF),^57^ Brief Resilient Coping Scale (BRCS)^58^ and the Trail Making Test (TMT).^59^ Daily app engagement was recorded, and participants completed an exit feedback questionnaire.

### Brain imaging

Brain imaging MRI scans were performed on a MAGNETOM Prisma 3 T scanner, configured with 64-channel receiver head coils (Siemens Healthcare, Erlangen, Germany), at the Ruth and Meir Rosental Brain Imaging Center (MRI), Reichman University. Due to dopaminergic effects on rsFC,^32^ Parkinson’s disease medications were administered 2 h prior to MRI scans. The MRI protocol included the following sequences: two runs of rs-fMRI scans (300 volumes, 9:28 min) were acquired using a multi-band echo planar imaging sequence, CMRR EPI 2D,^60,61^ scan parameters: repetition time (TR), 1870 ms; echo time (TE), 30 ms; flip angle, 75°; voxel size, 3.0 × 3.0 × 2.0 mm; field of view (FOV), 192; number of slices, 58; axial slices parallel to the anterior commissure - posterior commissure plane. During scanning, participants were asked to remain still and relaxed, with their eyes fixated on a cross, and without deliberately thinking of anything. Foam pads and earplugs were employed to reduce head motion and scanning noise. Structural T1-weighted MRI scans were acquired for co-registration purposes using a T1-weighted 3D magnetization-prepared rapid gradient-echo (MPRAGE) sequence in a sagittal plane with 1 mm isotropic resolution. The sequence parameters are TR, 2000 ms; TE, 1.9 ms; flip angle, 9°; inversion time, 920 ms; FOV, 256 × 256; and 176 contiguous slices. The MRI protocol also included T2-fluid-attenuated inversion recovery (FLAIR) sequences, using standard parameters for clinical brain evaluation.

### BOLD data preprocessing and seed selection

RsFC analysis was carried out using the CONN (RRID: SCR_009550, https://web.conn-toolbox.org, v22a), as implemented using the statistical parametric mapping software SPM12 (http://www.fil.ion.ucl.ac.uk/spm). Functional volumes preprocessing pipeline included realignment with correction of susceptibility distortion interactions, slice timing correction, outlier detection, direct segmentation and Montreal Neurological Institute-space normalization, with a resolution voxel size of 2.0 × 2.0 × 2.0 mm and spatial smoothing (8 mm full width at half maximum Gaussian kernel) steps.^62^ A component-based noise correction procedure (CompCor) approach^63^ was used to identify additional confounding temporal factors controlling for physiological noise, BOLD signal present in white matter and head motion effects. Residual BOLD time series were then bandpass filtered at a frequency range of 0.008–0.09 Hz. Individual connectivity maps were generated using the seed-to-voxel approach. Bivariate correlation analysis was used to determine the linear association of the BOLD time series between the seed and significant voxel clusters. Fisher’s Z transformation was applied to the correlation coefficients to satisfy normality assumptions. Finally, participants with head motions of >3 mm in any direction between volumes, rotations of >3° in any axis during scanning or mean framewise displacement (FD) scan-to-scan head motion > 0.5, in either the pre- or post-treatment maps, in both runs, were excluded from the dataset.

To elucidate the parallel neural mechanisms underlying the combined effects of standard levodopa treatment and the DopApp™ digital intervention on both motor and NMSs, we investigated segregated thalamocortical circuits. These circuits were chosen due to the thalamus’s role as a critical relay integrating BG output to the cortex.

To assess the impact of sensorimotor training, rsFC was evaluated using *a priori* seed regions located in key ventral lateral (VL) thalamic nuclei [ventral lateral anterior (VLa) and ventral lateral posterior (VLp)],^64^ as defined by the extended Human Connectome Project-MultiModal Parcellation Atlas (HCPex).^65^ The VLa primarily convey BG output to premotor and supplementary motor areas, while the VLp nucleus serves as the main thalamic relay of cerebellar output to the primary motor cortex (M1).^64,66^ Notably, VLp is interconnected with other thalamic nuclei and may be indirectly modulated by BG activity or influenced by BG dysfunction.^24^ Accordingly, as a hypothesis-driven analysis, we additionally examined functional connectivity alterations within a VL-M1 mask. These nuclei have been implicated in regulating Parkinson’s disease motor symptoms,^67,68^ and are critical for capturing network-level alterations in integrated motor circuit function.

To examine the limbic thalamocortical pathway, we selected *a priori* seed regions within thalamic subregions implicated in limbic processing, based on the HCPex atlas.^65^ These included bilateral seeds in the anteroventral thalamic nucleus (AV), the mediodorsolateral parvocellular nucleus (MDl) and the mediodorsomedial magnocellular nucleus (MDm). The AV nucleus, a principal component of the limbic thalamus, links memory and emotion circuits by interfacing with limbic regions of the default mode network (DMN), thereby supporting integrative information processing.^69-71^ The MD complex, through its extensive connections with the prefrontal cortex and other cortical areas, contributes to higher cognitive functions, including recognition memory and familiarity, via inputs from the perirhinal cortex, and plays a role in modulating cortical network dynamics.^69^ In addition to these BG seed regions and informed by our previous work involving digital interventions targeting emotion regulation,^39,40^ we included bilateral amygdala (Amyg) seeds to assess potential changes in functional connectivity associated with affective and emotional processing.

### Statistical analysis

Efficacy analyses were performed for all participants who had met the *a priori* analysis inclusion criteria of completing ≥17 of the 21-day treatment. Two-tailed Welch’s unequal variances *t*-tests were performed to compare continuous variables between groups when a normality assumption was held according to Kolmogorov–Smirnov tests (*P* < 0.05 was considered significant). Effect sizes were evaluated using Cohen’s *d* method. Categorical data were compared using chi-square/Fisher’s exact tests. The response rate analysis was calculated as the proportions in each group and was compared using Fisher’s exact test.

Sensitivity analyses were also performed for the primary therapeutic outcome, including (i) exclusion of outliers (defined as participants having >3 median absolute deviations from the group median); (ii) inclusion of all study subjects who signed the informed consent, were randomized, completed the baseline evaluation and commenced treatment (*n* = 42); (iii) non-parametric statistical tests using the Wilcoxon rank test; and (iv) a linear model with baseline MDS-UPDRS total score, number of years from Parkinson’s disease diagnosis and the daily levodopa equivalent dose^47^ included as covariates.

To assess the relationship between variables, Pearson correlation analyses were performed within the DopApp™ group. For each variable pair, scatter plots were generated and overlaid with a linear regression line and its 95% confidence interval. All analyses were performed using R Statistical Software (v4.4.1; R Core Team 2024) and Python (v3.13.1; Python Software Foundation, 2024).

### Determination of sample size

Given the exploratory and proof of concept (PoC) nature of this study, a target enrolment of 40 PwP was planned. Due to the absence of prior data on expected effect sizes and uncertainties regarding app usability, a formal *a priori* power calculation was not possible. Sensitivity analyses indicated that this sample size provided 80% power at a 5% significance level to detect a between-group difference greater than 5.5 points in the total MDS-UPDRS score, supporting the study’s ability to identify meaningful effects despite not being powered to detect the established minimal clinically important difference (MCID) of 6.7 points.^72^

### Imaging statistical analysis

At the group level, rsFC individual maps were analysed using the mixed design repeated measure analysis of variance (ANOVA) model to test the main interaction effect between time and group. Whole-brain rsFC analyses employed an exploratory threshold of *P* < 0.005 voxel-wise combined with cluster-level false discovery rate (FDR) correction at *P* < 0.05 and a minimum cluster size of 50 voxels. This approach balances sensitivity and specificity given the modest sample size and PoC design. Primary hypothesis-driven analyses within the motor network mask used a more stringent voxel-wise threshold of *P* < 0.001 with peak-level FDR correction (*P* < 0.05). A bivariate group-level whole-brain regression analysis with non-imaging covariates (e.g. psychological data and MDS-UPDRS) model was used to identify global brain correlations. Given the exploratory nature of the study and multiple clinical and engagement variables tested, no additional correction across these variables was performed.^73^ The analysis was implemented in SPM12 software (http://www.fil.ion.ucl.ac.uk/spm) with a parametric analysis approach across the entire brain volume.^62^ The Pearson correlation was then used to test for associations with the non-imaging covariates, where the REX toolbox was used to extract cluster connectivity values.^62^

## Results

### Patients

A total of 190 individuals responded to the study advertisement and answered the pre-screening questionnaire. Forty-two met the eligibility criteria and were subsequently enrolled and randomized to the study procedures. Of these, one participant from the DopApp™ group withdrew consent, and two participants from the placebo group were excluded due to major protocol deviations (Supplementary Fig. 2). Thirty-nine patients completed the study as per protocol (DopApp™ *n* = 20; placebo *n* = 19). The mean age of participants was 67.1 ± 6.7 years, 56% were male and Parkinson’s disease duration was 5.64 ± 3.1 years. Participant characteristics are shown in Table 1, with no significant between-group baseline differences. Neuroimaging was offered to all participants. Seven were ineligible, three withdrew the neuroimaging consent after the first scan and one was excluded for excessive, in-test, motion artefact. With no group differences in head motion (Supplementary Table 2), the neuroimaging sub-study included 31 participants (DopApp™ *n* = 17; placebo *n* = 14). Eight participants experienced an adverse event (AE) (DopApp™ *n* = 4; placebo *n* = 4), all of which were considered unrelated to the treatment protocol of DopApp™/placebo. One serious AE (dyspnoea requiring hospitalization) was observed in the placebo group.

**Table 1:** Baseline characteristics

	DopApp™ (*N* = 20)	Placebo (*N* = 19)	Total (*N* = 39)
Age, years	67.25 (6.7)	66.9 (7.0)	67.1 (6.7)
Male sex	11 (55%)	11 (58%)	22 (56%)
Parkinson’s disease duration, yrs	5.85 (3.2)	5.42 (3.2)	5.64 (3.1)
Hoehn and Yahr stage			
0	0 (0%)	0 (0%)	0 (0%)
1	8 (40%)	5 (26%)	13 (33%)
2	11 (55%)	14 (74%)	25 (64%)
≥3	1 (5%)	0 (0%)	1 (3%)
MDS-UPDRS total score	36.55 (12.5)	42.42 (18.6)	39.41 (15.8)
Presence of motor fluctuations	10 (50%)	13 (68%)	23 (59%)
Presence of dyskinesia	7 (35%)	6 (32%)	13 (33%)
Duration of levodopa treatment, years/months	3.1 (1.5)	2.8 (2.0)	2.9 (1.8)
Daily levodopa dose, mg (range)	526 (150–1460)	500 (250–1100)	513 (150–1460)
LEDD (range)	626 (300–1560)	692 (250–1475)	659 (250–1560)
Antiparkinsonian medication use			
Dopamine agonists	5 (21%)	6 (31%)	11 (28%)
MAO-B inhibitors	11 (55%)	15 (79%)	26 (66%)
Amantadine	7 (35%)	6 (31%)	13 (33%)
Exercise—days per week	5.4 (2.0)	4.5 (2.0)	4.9 (2.0)
Exercise—minutes per day	70.3 (39.5)	64.5 (30.5)	67.5 (35.1)
Questionnaires baseline			
MoCA	27.15 (2.2)	26.26 (1.8)	26.72 (2.1)
PDQ39	19.84 (12.2)	24.7 (15.2)	22.21 (13.8)
BDI-II	8.9 (7.1)	9.16 (6.6)	9.03 (6.8)

Data are mean (SD) or *n* (%).

LEDD, levodopa equivalent daily dose; MAO-B, monoamine oxidase B, MoCA, Montreal Cognitive Assessment; PDQ39, Parkinson’s Disease Questionnaire-39; BDI-II, Beck’s Depression Inventory II.

### Adherence to daily treatment

Daily treatment protocol adherence was high for both the DopApp™ and placebo groups, with completion rates of 95.5% and 93.5%, respectively, with no significant between-group differences (Supplementary Fig. 3A). In both groups, participants generally reported positive experiences with the app, particularly noting that it was easy to use and navigate. DopApp™ group participants reported a more positive experience when asked about whether the app met their expectations, contributed to their health and their overall satisfaction (Supplementary Fig. 3B). The DopApp™ group also showed high ratings on additional app-specific questions, highlighting satisfaction with the clarity of practices, perceived usefulness of the content, successful integration into daily routines and strong willingness to recommend the app to a fellow Parkinson’s disease patient, with 90% indicating they would do so (Supplementary Fig. 3C).

Overall, 64.1% of participants correctly identified their group assignment (DopApp™ 55%, placebo 73.7%). When testing whether this distribution differed significantly from what would be expected by chance (50%), the result was not statistically significant (chi-square test, *P* = 0.078), suggesting that the blinding procedure was effective, with only a marginal indication of unblinding. Additionally, there were no significant differences in guessing accuracy between the groups (*P* = 0.378), further supporting the blinding process’s relative success.

### Clinical outcomes

**Figure 2:**
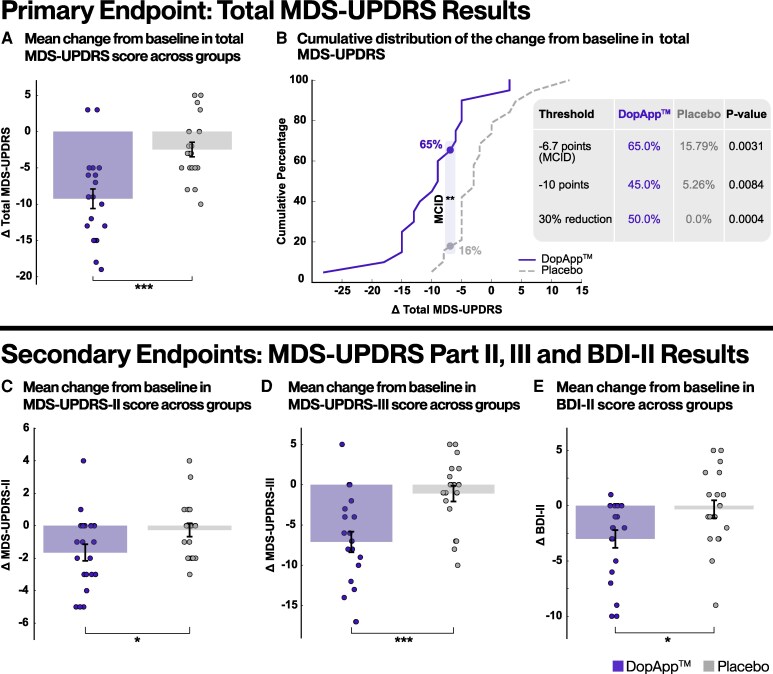
**DopApp™ treatment leads to significant improvements in motor and NMSs compared to placebo.** (**A**) Mean change from baseline to post-intervention in total MDS-UPDRS scores. Treatment with DopApp™ led to significantly greater improvement than placebo, with a treatment difference of 7.75 points [*t*(37) = 3.8; *P* = 0.0005; *d* = 1.22]. (**B**) Cumulative distribution of individual changes in total MDS-UPDRS scores, showing the proportion of participants in each group achieving various levels of improvement. The vertical dashed line indicates the MCID of 6.7 points reduction from baseline. The DopApp™ group showed a significantly greater proportion of responders across all thresholds (*P* < 0.01, Fisher’s exact test). (**C**) Mean change in MDS-UPDRS Part II scores (motor experiences of daily living). The DopApp™ group showed a significantly greater improvement (−1.75 ± 2.49) compared to placebo (−0.26 ± 1.79), yielding a treatment difference of 1.49 points favouring DopApp™ [*t*(37) = 2.13; *P* = 0.039; *d* = 0.68]. (**D**) Mean change in MDS-UPDRS Part III scores (motor examination). The DopApp™ group showed a significantly greater improvement (−7.35 ± 6.29) compared to placebo (−0.89 ± 4.68), yielding a treatment difference of 6.46 points [*t*(37) = 3.63; *P* = 0.0009; *d* = 1.16]. (**E**) Mean change in BDI-II scores. The DopApp™ group showed a significant reduction in depressive symptoms (−3.5 ± 5.02), while the placebo group exhibited a slight worsening (+0.05 ± 4.31), yielding a treatment difference with a medium effect size [*t*(37) = 2.36; *P* = 0.023; *d* = 0.76]. Bars represent group mean; error bars indicate SEM. Each dot represents data from an individual participant. MCID, minimal clinically important difference, (*P < 0.05; **P < 0.01; ***P < 0.001), DopApp™, *n* = 20; placebo, *n* = 19.

The study’s primary therapeutic outcome was the change in MDS-UPDRS total scores (sum of Parts I, II and III). Treatment with DopApp™ led to a significant improvement compared to placebo, with a mean ± SD change from baseline in MDS-UPDRS total score of −9.7 ± 7.03 in the DopApp™ group and −1.95 ± 5.57 in the placebo group (*P* = 0.0005; *d* = 1.22; Fig. 2A). Likewise, a significantly greater response rate was observed in the DopApp™ group across all response thresholds, as illustrated by the distinct cumulative distributions of the two groups (Fig. 2B; Supplementary Table 3). Specifically, 65% of the DopApp™ group exceeded the MCID of 6.7 points,^72^ whereas only 15.8% of the placebo group reached this threshold. Although the mean baseline MDS-UPDRS total score was slightly lower in the DopApp™ group, the difference was not statistically significant (*P* = 0.25), and results remained unchanged when accounting for baseline variability (see sensitivity analysis in Materials and methods and Supplementary Table 4). Further analysis of MDS-UPDRS Part II (intergroup difference: 1.49 points; *P* = 0.039; *d* = 0.68) and MDS-UPDRS Part III motor scores (intergroup difference: 6.46 points; *P* = 0.0009; *d* = 1.16) support the primary outcome (Fig. 2C and D). BDI-II depression scores improved by 3.5 ± 5.02 in the DopApp™ group, compared to a negligible change of +0.05 ± 4.31 in the placebo group, yielding a medium effect size (*P* = 0.023; *d* = 0.76) (Fig. 2E). All sensitivity analyses produced similar results with significant treatment differences (Supplementary Table 4). No significant intergroup differences were apparent for any other psychological, cognitive or Parkinson’s disease-related outcomes (Supplementary Table 5).

### Motor function correlates: clinical, neural and digital metrics

**Figure 3:**
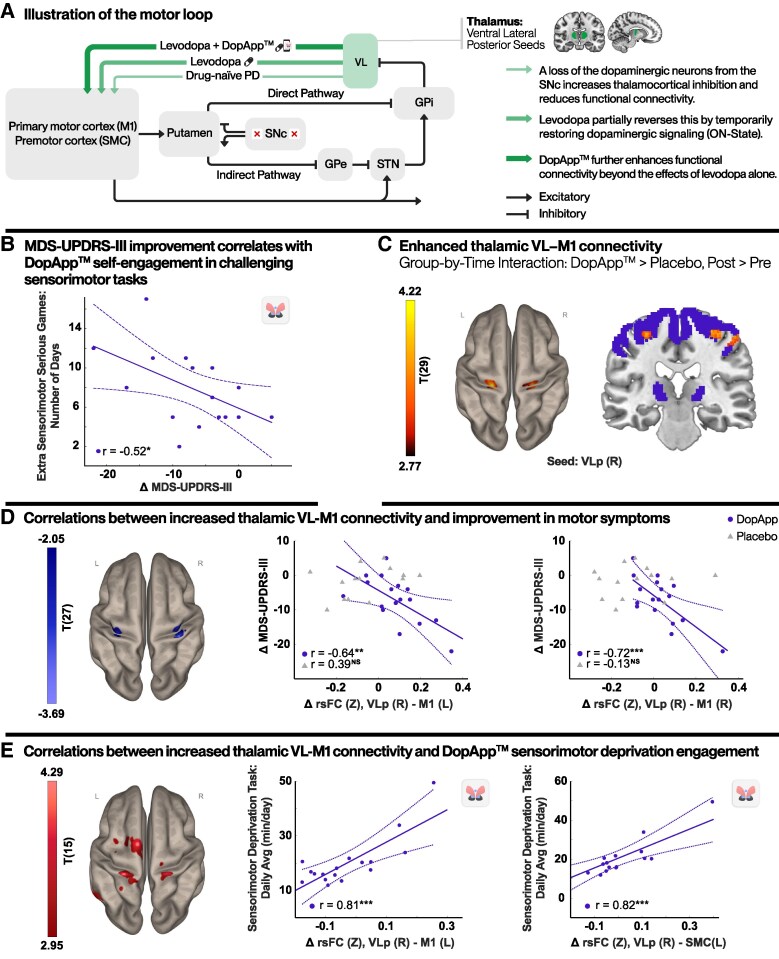
**Association between motor neuroplasticity, motor symptom improvement and sensorimotor task engagement.** (**A**) A simplified illustration of the BG motor circuit in Parkinson’s disease. Effects of treatment on thalamocortical transmission deficits. VL, ventral lateral thalamic nucleus, SNc, substantia nigra pars compacta, GPi, globus pallidus internus, GPe, globus pallidus externus, STN, subthalamic nucleus (**B**) Scatter plot depicting the relationship between changes in MDS-UPDRS III scores and usage consistency of sensorimotor serious games sessions (beyond the scheduled protocol). This correlation indicates that increased voluntary motor training is associated with greater motor improvement. (**C**) Seed-to-voxel group-by-time interaction ANOVA model analysis showing significantly increased rsFC between the right VLp seed and bilateral M1 regions in the DopApp™ group compared to placebo (DopApp™ > placebo, post > pre) within the thalamocortical mask, voxel-wise threshold of *P* < 0.001 with peak-level FDR correction (*P* < 0.05). Additional details are reported in Supplementary Table 6. (**D**) Correlations between changes in thalamocortical rsFC and motor symptom improvement. Left, a regression map with changes in MDS-UPDRS Part III scores covariate. Right, scatter plots depicting this relationship. No significant associations were found in the placebo group. (**E**) Correlations between thalamocortical rsFC alterations and the daily dose of sensorimotor-deprivation task. Left, a regression map with the daily dose (average daily sensorimotor-deprivation engagement) covariate. Right, scatter plots depicting these relationships, supporting a link between app daily dosage usage and motor-related neuroplasticity. Regression analysis maps: voxel level *P* < 0.005, cluster level *P* < 0.05, FDR corrected. Scatter plots: each dot represents data from an individual participant, the solid line denotes the linear regression fit and the curved lines indicate the 95% confidence interval, *r*, Pearson correlations value (*P < 0.05; **P < 0.01; ***P < 0.001), DopApp™, *n* = 17; placebo, *n* = 14. R, right, L, left. Brain images were generated using CONN (RRID:SCR_009550, https://web.conn-toolbox.org), v22a and MRIcroGL software (https://www.nitrc.org/projects/mricrogl). See also Supplementary Table 8 for more details.

Motor outcomes were further examined through associations with complementary data: app engagement metrics, based on sensorimotor serious games data, and thalamocortical motor circuit alterations (Fig. 3A). Engagement metrics included exposure (total engagement time), daily dose (average daily use) and usage consistency (number of active days). See Materials and methods for additional information. This multi-level approach revealed that improvements in MDS-UPDRS Part III were significantly correlated with greater usage consistency in serious sensorimotor game sessions (*r* = −0.52; *P* < 0.04; Fig. 3B), with no associations observed for emotion regulation components.

Next, we found that these improvements at the clinical level were accompanied by functional reorganization within motor-related brain circuits. We examined our hypothesis-driven region of interest analysis and focused on the motor VL thalamus nuclei and M1 to define significant areas for brain correlations. Figure 3C shows a significant group-by-time interaction within the VL-M1 circuit, with increased rsFC between bilateral M1 clusters (right: *T* = 4.42, *k* = 177; left: *T* = 4.01, *k* = 100, *P*_FDR_ < 0.05) and the right VLp seed within the thalamomotor mask. Importantly, similar results were obtained in whole-brain analysis showing a significant group-by-time interaction within the right VLp-M1 circuit (*T* = 4.55; *k* = 738; *P*_FDR_ < 0.01). Trial group analysis shows bilateral increased rsFC in this circuit (*T* = 4.42, 4.49; *k* = 260, 202; *P*_FDR_ < 0.01, right and left, respectively). No significant results were shown in placebo group analysis, Supplementary Fig. 4. See Supplementary Table 6 for additional results. In line with the group-by-time results, increased rsFC between the right VLp and bilateral M1 were significantly correlated with reduced MDS-UPDRS Part III scores (DopApp™: *r* = −0.72, −0.64; *P* < 0.001; *P* < 0.005; placebo: N.S, for the right and left M1 clusters, respectively; Fig. 3D).

Exploring the associations between motor circuit alterations and app-based usage metrics yielded that the daily dose of sensorimotor-deprivation tasks was strongly correlated with increased rsFC between the right VLp seed and both the M1 (*r* = 0.81; *P* < 0.001) and supplementary motor cortex (SMC) (*r* = 0.82; *P* < 0.001; Fig. 3E) in the DopApp™ group. Taken together, these results suggest that repeated engagement with sensorimotor-deprivation components contributed to functional reorganization within thalamocortical motor circuits. Additional significant results are listed in Supplementary Table 8.

### Non-motor function correlates: clinical, neural and digital metrics

**Figure 4:**
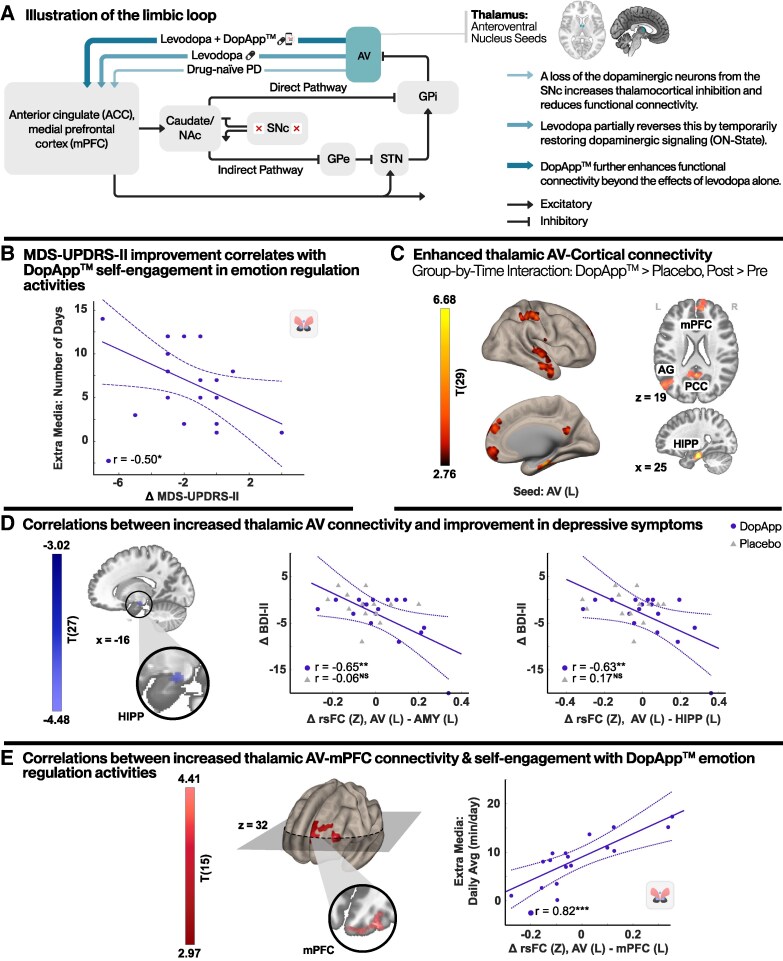
**Association between limbic neuroplasticity, NMS improvement and emotion regulation practice.** (**A**) Schematic illustration of the BG limbic circuit in Parkinson’s disease. Effect of treatment on thalamocortical transmission deficits. AV, anteroventral thalamic nucleus, NAc, nucleus accumbens, SNc, substantia nigra pars compacta, GPi, globus pallidus internus, GPe, globus pallidus externus, STN, subthalamic nucleus (**B**) Scatter plot depicting the relationship between change in MDS-UPDRS II and usage consistency of emotion regulation sessions (beyond the scheduled protocol). A significant negative correlation indicating that greater voluntary emotion regulation practice is linked to increased functional connectivity. (**C**) Seed-to-voxel group-by-time interaction ANOVA model analysis showing significantly increased rsFC between the left AV seed and cortico-limbic areas related to the DMN, memory and emotion control (DopApp™ > placebo, post > pre). (**D**) Correlations between changes in AV rsFC and changes in BDI-II scores (depressive symptoms). Left, a regression map with changes in BDI-II scores covariate. Right, scatter plots depicting these relationships in both the amygdala (AMY) and the hippocampus (HIPP). No significant associations were found in the placebo group. (**E**) Correlations between changes in AV rsFC and increased self-initiated daily dose of emotion regulation content. Left, a regression map with media daily-dose engagement covariate. Right, a scatter plot depicting this relationship in the mPFC, suggesting that higher daily dosage contributes to targeted neuroplastic changes. Regression analysis maps: voxel level *P* < 0.005, cluster level *P* < 0.05, FDR corrected. Scatter plots: each dot represents data from an individual participant, the solid line denotes the linear regression fit and the curved lines indicate the 95% confidence interval, *r*, Pearson correlations value (*P < 0.05; **P < 0.01; ***P < 0.001), DopApp™, *n* = 17; placebo, *n* = 14. R, right, L, left. DMN, default mode network; mPFC, medial prefrontal cortex; PCC, posterior cingulate cortex; AG, angular gyrus. Brain images were generated using CONN (RRID:SCR_009550, https://web.conn-toolbox.org), v22a. See also Supplementary Tables 7 and 8 for details.

Similar to the above motor analysis, non-motor outcomes were further examined through associations with complementary data: engagement with app-based emotion regulation media content and thalamocortical limbic circuit alterations (Fig. 4A).

Analysis of app-based emotion regulation metrics revealed that usage consistency (number of engagement days) with this content significantly correlated with improvements in MDS-UPDRS Part II (*r* = 0.50; *P* < 0.05; Fig. 4B). Since Part II is indirectly yet substantially influenced by NMSs,^74^ this association suggests that sustained engagement contributed to the observed improvements.

We then examined the thalamocortical pathway focusing on thalamic subdivisions associated with the limbic network to delineate regions exhibiting significant connectivity changes to guide targeted correlation analyses. Following intervention, a significant group-by-time interaction was demonstrated, with increased rsFC between the left AV with DMN nodes compared to placebo: medial prefrontal cortex (mPFC) (*T* = 4.34; *k* = 230; *P*_FDR_ < 0.05), posterior cingulate cortex (PCC) (*T* = 4.15; *k* = 237; *P*_FDR_ < 0.05) and left angular gyrus (AG) (*T* = 4.19; *k* = 346; *P*_FDR_ < 0.05). Additionally, a group-by-time interaction within the MTL–AV pathway was observed, specifically in bilateral clusters localized in the hippocampus (*T* = 4.36, 3.46; *k* = 89, 215; *P*_FDR_ < 0.05) and in the para-hippocampus (*T* = 6.67, 2.91; *k* = 313, 38; *P*_FDR_ < 0.05) compared to placebo (Fig. 4C; Supplementary Table 7).

In line with the group-by-time results, we also found significant correlations between reductions in BDI-II depression scores and increased rsFC between the left AV nucleus and both the left amygdala and left hippocampus (*r* = −0.65, *P* < 0.005; *r* = −0.63, *P* < 0.007, respectively, placebo: N.S; Fig. 4D). Regarding app usage, increased rsFC between the left AV and the mPFC (BA9, 10) was correlated with higher self-initiated daily doses of emotion regulation content usage (*r* = 0.82; *P* < 0.001; Fig. 4E). This neural pathway, which is part of the DMN, is implicated in self-referential processing and emotional regulation. Importantly, no significant relationship was found between changes in VL, the motor thalamic nuclei, connectivity and emotion regulation activity measures, supporting the hypothesis of distinct and parallel neural pathways underlying the intervention’s motor and emotional components. Additional significant results are listed in Supplementary Table 8.

### Amygdala network correlates: clinical, neural and digital metrics

**Figure 5:**
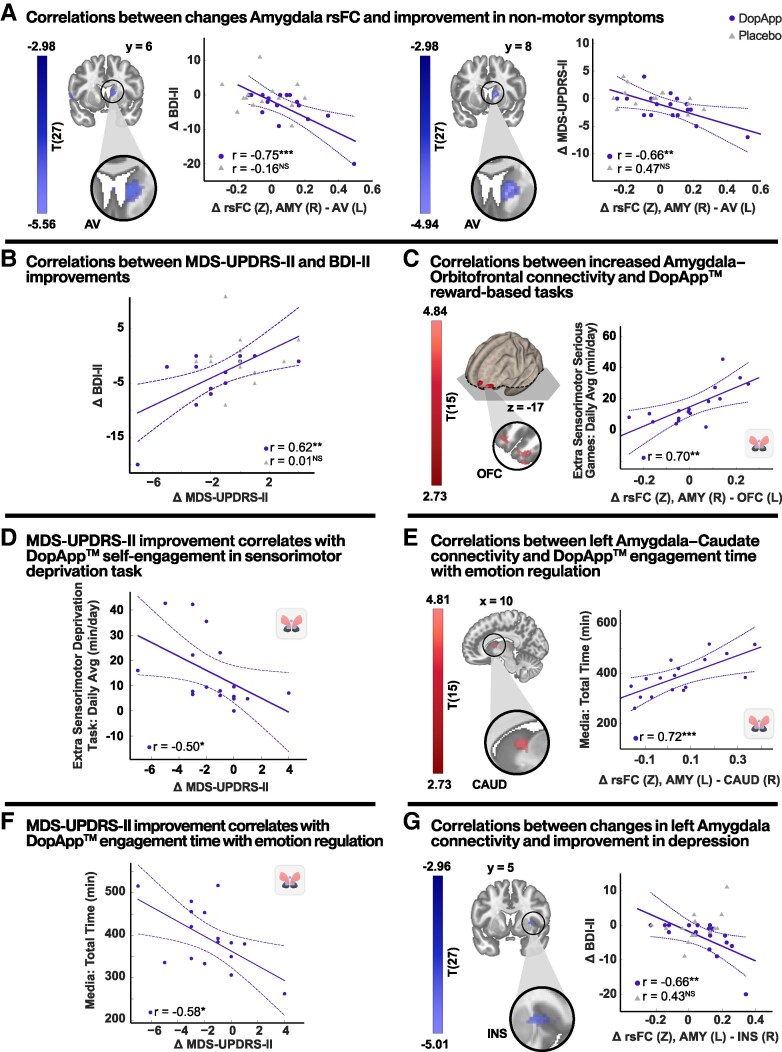
**Association between amygdala neuroplasticity, NMS improvement and engagement with DopApp™.** (**A**) Correlations between changes in amygdala (AMY) rsFC and changes in psychological state: left, a regression map with changes in BDI-II scores covariate. The corresponding scatter plot depicts relationship in cluster peaks in the AVs. Right, a regression map with changes in MDS-UPDRS II scores covariate. The corresponding scatter plot depicts relationship in cluster peaks in the right AV. No significant associations were found in the placebo group. (**B**) A scatter plot depicting the relationship between changes in MDS-UPDRS Part II and BDI-II scores in the DopApp™ and placebo groups. A significant positive correlation indicating that greater improvements in motor experiences of daily living are associated with greater reductions in depressive symptoms. (**C**) Correlations between increased OFC connectivity and reward-based tasks. Left, a regression map with daily-dose measures of reward-based games covariate. Right, a scatter plot depicting relationships in the cluster peak in the right OFC. This neural pathway is well established in supporting motivation and reward-based decision-making. (**D**) A scatter plot showing a significant correlation between self-initiated daily dose of the sensorimotor task and improved MDS-UPDRS II scores, suggesting that behavioural engagement contributes to motor improvement. (**E**) Correlations between changes in the left amygdala rsFC and app usage: Regression map with total exposure to emotion regulation content covariate and the corresponding scatter plot depicting relationships in cluster peaks in the left caudate (CAUD) area. (**F**) A scatter plot showing changes in MDS-UPDRS II scores versus the total exposure to emotion regulation content. (**G**) Correlations between changes in left amygdala rsFC and improvement in depression: a regression map with changes in BDI-II scores covariate and the corresponding scatter plot depicting relationship in cluster peaks in the right insula (INS) area. Regression analysis: voxel level *P* < 0.005, cluster level *P* < 0.05, FDR corrected. Scatter plots: each dot represents data from an individual participant, the solid line denotes the linear regression fit and the curved lines indicate the 95% confidence interval, *r*, Pearson correlations value (*P < 0.05; **P < 0.01; ***P < 0.001), DopApp™, *n* = 17; placebo, *n* = 14. R, right, L, left. Brain images were generated using CONN (RRID:SCR_009550, https://web.conn-toolbox.org), v22a. See also Supplementary Table 8 for details.

Testing the amygdala seeds, no group-by-time interaction effects were observed. Nevertheless, significantly increased post-intervention connectivity was found in the DopApp™ group between the left amygdala and the left caudate and between the right amygdala and the right thalamus (*P*_FDR_ < 0.05; Supplementary Fig. 5). Importantly, significant associations were found between rsFC and both psychological state measures and related app engagement measures. In the DopApp™ group, increased rsFC within the right amygdala–AV pathway was correlated with improvements in both BDI-II (*r* = −0.75; *P* < 0.001) and MDS-UPDRS Part II (*r* = −0.66; *P* < 0.005) scores, while no significant correlations were found in the placebo group (Fig. 5A). Notably, a significant correlation was also observed between changes in BDI-II and MDS-UPDRS Part II scores (*r* = 0.62; *P* < 0.008; Fig. 5B). These findings, along with the involvement of the AV, as a limbic subregion of the thalamus, further support the interpretation that the MDS-UPDRS Part II measure is influenced by emotional NMS in Parkinson’s disease.

The right amygdala network was also implicated in motivational processes, as illustrated in Fig. 5C. To quantify this, we analysed the self-initiated sensorimotor serious games measures, which reflect involvement in cognitively demanding and reward-based tasks. We found that the higher daily dose of this measure was correlated with increased rsFC between the right amygdala and the orbitofrontal cortex (BA 11) (*r* = 0.72; *P* < 0.001), in contrast to the rsFC patterns observed with self-initiation with emotion regulation activities (Fig. 5C). The amygdala–orbitofrontal (OFC) pathway is well established in supporting motivation and reward-based decision-making.^75^ Notably, self-initiated daily doses of the sensorimotor-deprivation task, the most demanding of the reward-based components, were specifically correlated with improvements in MDS-UPDRS Part II scores, suggesting a link between self-motivation and improvements in subjective perception of motor function (*r* = −0.5; *P* < 0.04; Fig. 5D).

Exploring associations within the left amygdala network revealed significant correlation between increased rsFC in the left amygdala–caudate pathway and the total exposure to emotion regulation content (*r* = 0.72; *P* < 0.001; Fig. 5E). Interestingly, this usage measure was also correlated with improvements in MDS-UPDRS Part II scores (*r* = −0.58; *P* < 0.02; Fig. 5F). Additionally, we found that increased rsFC in the left amygdala–insula pathway was correlated with the extent of BDI-II score reductions (*r* = −0.66; *P* < 0.005; Fig. 5G). Additional data is provided in Supplementary Table 8.

## Discussion

In this study, we demonstrate that 3 weeks of using a multimodal digital intervention (DopApp™) alongside standard levodopa therapy significantly improved motor and non-motor outcomes in PwP. These effects were associated with parallel modulation of rsFC within the thalamocortical motor and limbic networks. Moreover, increased engagement with sensorimotor and emotion regulation activities was associated with both enhanced therapeutic effects and rsFC alterations, suggesting a digital dose–response relationship and task-specific neural plasticity. These results provide preliminary evidence that digitally delivered, behaviourally targeted interventions can augment dopaminergic pharmacotherapy benefits by promoting brain functional reorganization and may offer a scalable method to extend established Parkinson’s disease treatment efficacies.

Consistent with our first hypothesis, DopApp™ led to both statistically significant and clinically meaningful improvements in MDS-UPDRS total scores among levodopa-treated PwP, primarily driven by changes in Part III: Motor Examination.^72^ While the magnitude of this effect is larger than we initially anticipated for a short intervention, it is consistent with prior reports of short-term improvements following intensive, multimodal interventions in Parkinson’s disease. Specifically, accumulating evidence showing similar magnitudes of improvement have been documented in multidisciplinary team-based care and intensive rehabilitation programmes.^76-78^ For example, a recent large study involving over 1200 patients undergoing Parkinson’s Disease Multidisciplinary Complex Therapy (PD-MCT) demonstrated clinically meaningful improvements of ∼7.8 points in MDS-UPDRS Part III after 14–20 days of inpatient treatment, with an estimated daily average slightly exceeding 1 h of therapy.^78^ These findings support the plausibility of our observed effects within a similar timeframe and the comparable intensity of therapeutic engagement time (see Supplementary Table 1). Several factors likely contributed to these rapid and robust therapeutic effects. DopApp™’s multidimensional design, targeting cognitive, emotional, sensorimotor and motor domains, aligns with current concepts in lifestyle medicine^79^ and clinical guidelines for comprehensive Parkinson’s disease care.^5-7^ The app also utilizes sensorimotor-deprivation and neuroscience elements, previously shown to enhance neuroplasticity,^42,48,80^ and brief, gamified tasks helped sustain user engagement and high adherence.^81,82^ This shows that digital intervention may complement standard dopaminergic treatment by augmenting therapeutic effects across motor and NMSs.

In addition to the motor improvements, we also observed improvements in both BDI-II scores and MDS-UPDRS Part II (Fig. 2). Given the low baseline BDI-II scores, we observed a statistically significant improvement in subclinical depressive symptoms. As depression is a key NMS of Parkinson’s disease, this result aligns with prior evidence supporting the potential of digital non-pharmacological interventions to enhance mood in PwP.^83-85^ However, the intervention’s effect on clinical depression remains to be established and should be investigated in future studies involving participants with higher baseline depressive symptom severity. MDS-UPDRS Part II is formally defined as an assessment of motor experiences of daily living rather than NMS. However, this is a patient-reported measure, and accumulating evidence indicates that self-rated activities of daily living are influenced not only by motor deficits but also by day-to-day symptom fluctuations as well as emotional and cognitive factors.^74,86-88^ Consistent with this, we found that improvement in MDS-UPDRS Part II was significantly correlated with improvement in BDI-II, suggesting that changes in affective state contributed, at least in part, to patients’ perceptions of functional change. This observation also aligns with prior report demonstrating discrepancies between patient-reported and clinician-rated outcomes and showing that NMSs correlate more strongly with MDS-UPDRS Part II scores than objective motor assessments.^87^ Taken together, these findings indicate that MDS-UPDRS Part II, while motor focused, also functions as a subjective functional measure that is substantially modulated by NMS, particularly in early Parkinson’s disease where current clinical scales show limited sensitivity.^86,89^ Notably, these effects were achieved without therapist involvement, relying entirely on smartphone-based self-engagement, distinguishing this intervention from prior approaches.^83-85^

Consistent with our second hypothesis, the intervention modulated motor thalamocortical connectivity. Engagement with sensorimotor-deprivation modules that utilize multisensory spatial memory tasks was associated with reduced MDS-UPDRS Part III scores and with corresponding connectivity alterations (Fig. 3). Prior studies also show impaired thalamocortical motor connectivity in Parkinson’s disease and improvements following therapy.^69-71^ Given that spatial learning deficits in Parkinson’s disease relate to both motor and NMSs,^90,91^ the observed effects may reflect the combined impact of targeted training on motor function and broader network integration. Additionally, while thalamocortical projections are predominantly ipsilateral,^92^ we observed enhanced bilateral connectivity in the DopApp™ group compared to the placebo group. Several mechanisms may account for this. Firstly, the cerebello-thalamocortical loop is double crossed, creating apparent contralateral pathways.^93,94^ Secondly, interhemispheric synchrony via the corpus callosum maintains phase-locked M1 regions.^95,96^ Thirdly, a small subset of VL thalamic neurons project contralaterally.^97^ These mechanisms may reflect compensatory processes reconciling ipsilateral wiring with strong contralateral motor correlations, consistent with neuroplastic compensatory patterns seen in stroke and ageing.^98,99^

Regarding our third hypothesis, we observed widespread connectivity increases between the AV thalamic nucleus and limbic and DMN regions, including the hippocampus, para-hippocampus, mPFC, PCC and AG (Fig. 4). The AV, a core node of the hippocampal–anterior thalamic axis, is critically involved in memory and emotional regulation^69-71^ and is implicated in Parkinson’s disease-related apathy, anxiety and cognitive decline.^100,101^ Functional disruptions of this nucleus have been reported in both Parkinson’s disease and Alzheimer’s disease, contributing to deficits in memory and emotion.^102^ In the present study, enhanced AV–DMN coupling suggests improved integration within limbic processing circuits,^45^ and increased coupling with MTL structures, including bilateral hippocampi and para-hippocampi, further supporting the AV’s role in modulating memory and affective function.^100^ Furthermore, greater exposure to psychological content was correlated with improvements in emotional and self-perceived motor function, alongside altered limbic–DMN connectivity. The absence of such associations with motor thalamic (VL) connectivity supports these effects’ circuit specificity.

Contrary to our hypothesis, shaped by our prior work,^39,40^ we did not observe a significant group-by-time interaction involving the amygdala. However, increased connectivity within the amygdala–striatum–thalamus pathway was observed in the DopApp™ group. Notably, this increase was significantly correlated with improvements in BDI-II and MDS-UPDRS Part II scores in the DopApp™ group, but not in the placebo group (Fig. 5). These results suggest meaningful individual-level effects, despite low baseline psychopathology levels in this cohort.^56^

Quantifying the efficacy of digital interventions remains a major challenge, and identifying dose–response relationships is critical for establishing therapeutic validity and optimizing treatment protocols.^46^ Here, we examined potential dose–response dynamics using app engagement metrics, including overall exposure, daily dose and usage consistency. These metrics reflect real-world usage and help identify effective engagement thresholds. For instance, increased exposure to psychological content beyond a certain time threshold was associated with enhanced rsFC, suggesting a usage efficacy threshold. Moreover, participants’ preferences for specific activity types (e.g. reward-based versus relaxation-focused) were linked to distinct patterns of rsFC alterations. These findings indicate that different content types may require different effective dosages to drive neural changes, highlighting the need for personalized therapeutic pathways. Overall, this underscores the importance of targeting and tailoring digital intervention components, both in type and dose, to specific clinical and neural outcomes.

Another important aspect of this study is its placebo-controlled design, addressing the unique challenge of developing a credible digital placebo that replicates engagement without therapeutic elements.^16,17^ Although blinding held statistically, results approached the conventional threshold, suggesting a potential marginal unblinding effect. Still, improvements observed in the placebo arm are consistent with a placebo effect, further supporting effective blinding. Future investigations should aim to optimize placebo design to minimize unblinding risk. Additionally, including a heterogeneous cohort spanning a broad range of disease severities and ages enhances the findings’ ecological validity and generalizability. Reliance on widely available standard smartphones, including among older adults,^103,104^ eliminates the need for specialized hardware, thereby enhancing the intervention’s scalability and clinical feasibility, particularly in resource-constrained settings. Importantly, despite a mean age of 67 and the presence of motor impairments, adherence rates were high, indicating that neither digital literacy nor physical limitations posed substantial barriers in this population. Finally, as a non-pharmacological intervention, DopApp™ is devoid of drug-related adverse effects, offering a favourable safety profile for individuals with Parkinson’s disease.

This study has several limitations. This short duration pilot study, conducted at a single centre with a relatively small sample size and without interim or long-term follow-up assessments, limits the generalizability and understanding of the durability and time course of intervention effects. These factors may also account for the lack of statistical significance in some of the outcomes, despite overall group-level improvements in the DopApp™ arm versus placebo. Additionally, the study design does not allow disentangling the specific contributions of the different components (psychological, sensorimotor, etc.), though distinct correlations were observed for each. Future studies could include component-specific arms to clarify individual effects. MDS-UPDRS Part III assessments were performed in the ON state based on patient report, without strict standardization of the time since the last levodopa dose, although within-subject assessment timing was kept consistent across visits. Future studies should incorporate more rigorous control of medication timing. Another limitation is the absence of objective cognitive assessments due to the focus on motor and psychological outcomes and the limited study duration. Cognitive complaints are a core component of Parkinson’s disease NMS,^105^ and future studies should incorporate standardized cognitive evaluations to better characterize the intervention’s impact in this domain. In addition, variability in rsFC findings across Parkinson’s disease studies, driven by differences in study design, imaging parameters and region-of-interest definitions, limits direct comparability with previous work. Finally, higher engagement was linked to better outcomes. This may reflect unmeasured factors, such as baseline motivation or reverse causality, with early gains driving continued use. As engagement was not predefined, causal claims remain limited. Still, correlations between engagement and rsFC in targeted brain networks suggest a possible underlying mechanism to be explored in future studies. Larger, multicentre studies with standardized imaging protocols and longer follow-up periods are needed to validate and extend the neural findings observed here. Expanding the DopApp™ platform to support long-term engagement, potentially through the integration of personalized interventions and generative artificial intelligence, represents a promising avenue for future development.

In conclusion, this study provides preliminary clinical and neurobiological evidence that a targeted, digital therapeutic can augment standard dopaminergic treatment in Parkinson’s disease. By engaging multidimensional digital activities, DopApp™ improved motor function, mood and daily living while modulating thalamocortical connectivity in motor and limbic circuits. Dose–response relationships between engagement and outcomes suggest that adaptive, task-specific digital tools may drive circuit-selective plasticity. These findings support the potential of scalable, adjunctive digital interventions and their integration into precision, drug-digital treatment strategies for neurodegenerative diseases. Furthermore, they represent an early step towards the development of hybrid digital-drug interventions that may personalize care, enhance pharmacological efficacy and extend therapeutic reach beyond the traditional clinical setting.

## Supplementary Material

fcag148_Supplementary_Data

## Data Availability

Anonymized data may be shared upon reasonable request to the senior author by qualified investigators for non-commercial use, subject to participant consent and applicable data protection legislation. The R Statistical Software and MATLAB/CONN scripts used in this study employed only standard, publicly available functions and packages; no custom code was generated.
