# Supplementary material for: Neural correlates of Parkinson’s improvements after combined digital-levodopa therapy: a pilot study
Source: Brain Commun. 2026 May 20;8(3):fcag148. doi: 10.1093/braincomms/fcag148 (PMC13188156; doi:10.1093/braincomms/fcag148)
Supplement: fcag148_Supplementary_Data [file fcag148_supplementary_data.pdf]

Neural correlates of Parkinson's improvements after combined digital-levodopa therapy: a pilot study

Supplementary Material

Table of Content

TABLE OF CONTENT **1**

1. ELIGIBILITY CRITERIA **2**

1.1 INCLUSION CRITERIA 2
1.2 EXCLUSION CRITERIA 2
1.3 ENTRY CRITERIA/DIAGNOSIS FOR MRI ASSESSMENT 3
1.4 PROHIBITED MEDICATIONS AND TREATMENTS 3

2. THE DOPAPP INTERVENTION PROTOCOL **4**

3. STUDY OUTCOMES **7**

4. SUPPLEMENTARY TABLES **9**

SUPPLEMENTARY TABLE 1: MEAN DAILY ENGAGEMENT TIME WITH DOPAPP 9
SUPPLEMENTARY TABLE 2: MAX HEAD MOTION 9
SUPPLEMENTARY TABLE 3: RESPONSE RATE ANALYSIS ACROSS MULTIPLE CLINICAL THRESHOLDS 9
SUPPLEMENTARY TABLE 4: PRIMARY OUTCOME MDS-UPDRS TOTAL SCORE SENSITIVITY ANALYSES 10
SUPPLEMENTARY TABLE 5: CHANGE FROM BASELINE IN EXPLORATORY OUTCOMES 11
SUPPLEMENTARY TABLE 6: LONGITUDINAL CHANGES IN CONNECTIVITY MEASURES: MOTOR CIRCUIT 13
SUPPLEMENTARY TABLE 7: LONGITUDINAL CHANGES IN CONNECTIVITY MEASURES: LIMBIC CIRCUIT 14
SUPPLEMENTARY TABLE 8: SIGNIFICANT ASSOCIATIONS BETWEEN ALTERATIONS IN BRAIN CONNECTIVITY 15

5. SUPPLEMENTARY FIGURES **17**

SUPPLEMENTARY FIGURE 1: DOPAPP™ SAMPLE SCREENS 17
SUPPLEMENTARY FIGURE 2: STUDY FLOWCHART 18
SUPPLEMENTARY FIGURE 3: ADHERENCE AND SATISFACTION SURVEY RESULTS 20
SUPPLEMENTARY FIGURE 4: THALAMOMOTOR CIRCUIT CONNECTIVITY ANALYSIS 20
SUPPLEMENTARY FIGURE 5: POST-INTERVENTION ALTERATIONS IN THE DOPAPPTM GROUP 21

SUPPLEMENTARY REFERENCES **22**

1. Eligibility Criteria

1.1 Inclusion criteria

For subjects to be eligible to participate in this trial, the following criteria must be met:

1. Male or Female subjects, aged 45-80.
2. Proficient in Hebrew reading and listening comprehension (the DopApp user interface is in Hebrew).
3. Confirmed diagnosis (by a letter from treating physician) of idiopathic Parkinson's disease (PD).
4. UPDRS section 3.4 (Finger tapping) score >=1 OR UPDRS section 3.1 (Speech) score >=1*.
5. Preserved activities of daily living: Study subjects should be able to walk, sit and stand without assistance, and own and be able to use their smartphone. They are additionally not wheelchair-bound and have good hearing and vision, naturally or corrected.
6. The study subjects are treated with levodopa containing medication (either immediate or controlled release), at a total daily dose of between 150mg and 1500mg, taken by no more than five daily administrations. The study subject's PD drug treatment dosing regimen has been stable for the last 30 days before study entry date and is not projected to be changed during the study period.
7. The subjects are able and willing to meet all protocol requirements, present themselves at the study site for all study visits and perform all tasks and tests included in the study protocol.

* Parkinson's disease presents with variable motor and speech symptoms. Therefore, these parameters were chosen to ensure inclusion of patients with measurable impairment in the domains targeted by the intervention. This approach balances scientific relevance and feasibility by allowing eligibility based on impairment in either domain, facilitating recruitment of a representative sample while maintaining sensitivity to detect meaningful changes.

1.2 Exclusion criteria

A subject will be excluded from the trial in the following circumstances:

1. Known to have another, concurrent and active, neurological disease, or is known to have a significant neurological disease other than PD that may affect motor function and cognition. Examples include, but are not limited to: multiple sclerosis, epilepsy, Huntington's disease, amyotrophic lateral sclerosis (ALS), cerebral palsy, and stroke.
2. Participant is using Duopa/Duodopa, has a history of brain surgery, deep brain stimulation, a significant head injury causing loss of consciousness greater than 30 minutes, or hospitalization due to a brain injury.
3. Participants have a known history of a clinically significant intracranial abnormality (e.g., stroke, hemorrhage, space-occupying lesion) that could account for the observed symptoms (excluding abnormalities consistent with underlying PD)
4. Previously undergone focused brain irradiation for any reason.
5. Participant has a history of suicidality in the last year.
6. Known, previously treated or untreated, and/or active central nervous system (CNS) metastases and/or carcinomatous meningitis.
7. Any relevant medical, surgical, or psychiatric condition, laboratory value, or concomitant medication which makes the subject unsuitable for study entry or potentially unable to complete all aspects of the study under the investigator's discretion.

8. Has regularly used opioids, which include, but are not limited to morphine, codeine, oxycodone, hydrocodone, fentanyl, tramadol, and methadone, more than three times per week in the 15 days prior to enrollment.
9. At screening, is a known regular user (including "recreational use") of any illicit drugs or has a history within the last year of active substance abuse including (but are not limited to): alcohol, cocaine, crack cocaine, ecstasy, hallucinogens (such as LSD, PCP, mushrooms, and salvia), heroin, inhalants (such as spray paints, markers, cleaning supplies), ketamine, meth, etc.
10. Known to have a neoplastic disorder, which is either currently active or has been in remission for less than one year.
11. Pregnant
12. Inability to sign the informed consent form
13. Subject has participated in another study of an investigational medicinal product (IMP) or a medical device within the last 30 days or is currently participating in another study of an IMP or medical device.
14. Does not own a smartphone.

1.3 Entry criteria/diagnosis for MRI assessment

1. Subject age is ≤76
2. Right-handed only
3. The subject is able and consents to undergo MRI scans.
4. The subject experiences dyskinesia of the head and neck less than 50% of the day.
5. Does not have a condition which is contra-indicated with MRI (metal implants, claustrophobia etc. according to the MRI safety form).

1.4 Prohibited medications and treatments

Subjects are not eligible to participate in this study if they received the following treatments within 30 days prior to entry into the study (Study Visit 1a), unless differently specified. In addition, these medications are prohibited to be administered to study subjects also during the conduct of the study. If treatment with these medications/therapies must be initiated after the subject has already entered the study, the subject should be withdrawn from the study ASAP:

1. Chronic treatment with anti-depressants, including SSRIs, SNRIs, NDRIs, TCAs is not allowed if their type and/or dosing regimen has been initiated and/or changed within a period of 30 days prior to study entry. It is also not permitted to change the type and/or dosing regimen of these drugs during the trial.
2. Chronic or frequent (more than three times per week) use of anxiolytic drugs, such as (but not limited to) benzodiazepines, except if taken at bedtime only.
3. Frequent (more than three times per week) use of opioids, which include, but are not limited to morphine, codeine, oxycodone, hydrocodone, fentanyl, tramadol, and methadone.
4. Treatment with non-pharmacological treatments for PD included in the app (speech, etc.) for a period of 30 days prior to study entry and during the trial.
5. Use of drugs such as barbiturates and St. John's wort that are known to affect metabolism of PD drugs

2. The DopApp intervention protocol

General

Each daily self-training session was originally designed to last approximately 30 minutes (excluding walking) and comprised the daily components described below. Importantly, all activities were also available on demand via a content library, allowing participants to engage in additional training beyond the standard daily protocol.

Technical requirements

The study version of DopApp was compatible with standard smartphones, including iPhone SE and iPhone 11–15 devices running iOS 17–18, as well as Samsung Galaxy A33 or newer devices running Android OS (version 12 or higher). No additional hardware-specific limitations were identified beyond standard smartphone requirements.

Daily protocol

DAY	NAME OF INTERVENTION	EXPLANATION
ALL	Each day, the activities included the following: maze game (a multisensory, gradual visual masking spatial navigation game), amplitude game (part of speech therapy, designed to train increasing loudness), pitch game, (part of speech therapy, designed to train pitch range), typing Game (part of fine-motor training), reading Game (part of speech therapy, design to train improved clarity of speech), whack-a-mole Game (part of fine-motor training, the participant has to quickly respond to appearing targets and repress response to distractors),Simon game (a game combining fine motor and auditory memory training, where the participant has to recall a sequence of sounds and enter it) and walking encouragement (participants were reminded to walk for at least 15 minutes daily).	
1	**Program introduction**	A brief introduction of the program
	**Setting medication reminders**	
	**Walking activity**	Set up
	**Speech therapy introduction**	An overview of the purpose of speech therapy.
	**Vocal warm-up**	Explanation
	**Setting volume target intensity**	
	**Amplitude**	Tutorial and game
	**Vocal range**	
	**Pitch**	Tutorial and game
	**Explanation of reading practice**	
	**Reading**	Tutorial and game
	**Maze**	Tutorial and game
	**Introduction of fine motor skills**	Describe how fine motor skills contribute to various tasks and activities.
	**Typing**	Tutorial and game
	**Whack**	Tutorial and game
	**Simon**	Tutorial and game
2	**About the app**	A brief explanation of the application's daily operating schedule and the planned activities.<br>through psychoeducation.
	**Muscle relaxation**	A brief video outlining the concept of progressive muscle relaxation
	**Muscle relaxation practice**	Audio
	**effective vocal practice**	A brief video demonstrating how to practice voice and speech training.
	**Toilet paper folding practice**	A fine motor exercise
3	**Traditional medicine and the app**	A brief video explaining about the relationship between traditional medicine and the app

	**Guided imagery**	A brief video introducing and explaining guided imagery as a therapeutic tool
	**Guided imagery to a safe place**	Audio
	**Neuroplasticity**	A brief video about Neuroplasticity
**4**	**Motivation**	A brief video describing how the disease impacts the motivational system, the ability to take initiative, follow through with beneficial actions, and maintain engagement.
	**Guided Imagery for relaxation**	Audio
	**Multi-senses**	A brief video about multi-senses
	**Tips for optimal movement**	A video describing three tips for optimal movement
**5**	Coping with stress and anxiety	
	**Breathing practice explanation**	A brief video demonstrating how breathing exercises can be used to manage and reduce stress.
	**About diaphragmatic breathing**	
	**Practice diaphragmatic breathing**	Interactive breathing video
	**Coordination practice**	Alternating number marking
	**About Neuro-dance**	A video demonstrating how dance can serve as a therapeutic tool.
	**Neuro-dance - fast paced movement**	
**6**	**Guided imagery for medication intake**	Explanation and practice
	**Mindful eating**	Audio
	**Practicing fine motor skills**	Ball exercise
**7**	**Week 1 Summary**	
	**Box breathing interactive breathing**	Interactive activity
	**Coordination**	Finger movement sequences
8	**Body Scan**	A brief video explaining a technique for directing our attention to different body parts proven to enhance sleep, memory, and more.
	**Body Scan practice**	Audio
	**Writing exercise**	explanation and practice
**9**	**Thoughts and their interpretation**	Explain how our thoughts influence our perception of reality and behavior, and how becoming aware of them can improve our wellbeing.
	**Activity choices**	Progressive muscle relaxation/diaphragmatic breathing/guided imagery to a safe place
	**Coordination**	movement sequences with rhythm
**10**	**Cognitive biases**	Video explaining cognitive biases
	**Activity choices**	Body scan/box breathing/guided imagery for relaxation
	**Toilet paper folding practice**	A fine motor exercise
	**Physiotherapy - stretches**	Improving motor skills.
**11**	**Balanced thoughts explanation**	An explanation of the steps to take after recognizing an extreme thought.
	**Balanced thoughts activity**	Interactive activity
	**Physiological sigh practice**	A brief introduction with a new breathing technique based on diaphragmatic breathing.
	**Activity choices**	Box breathing/diaphragmatic breathing practice
	**Fine motor skills**	Practicing with a pen
	**Physiotherapy**	Motor skills practice 1
**12**	**Breathing awareness**	Explanation and practice
	**Alternating number marking**	
	**Neuro-dance - balance**	

**13**	**Parable of the two arrows**	A short video explaining the parable of the arrows, which explores the question: Can we experience less suffering in response to life's challenges?<br>Body scan/breathing awareness practice
	**Practicing fine motor skills**	Ball exercise
**14**	**Week 2 summary**	
	**White bear effect**	Video
	**Activity choices**	Diaphragmatic breathing/guided imagery for relaxation/guided imagery for medication intake practice
	**Coordination**	Finger movement sequences
	**Physiotherapy**	Motor skills practice 2
**15**	**Struggle switch**	A short video explaining the "struggle switch" – our natural response to unpleasant emotions is to resist, but accepting these feelings can help reduce their impact.
	**Activity choices**	Body scan/box breathing/progressive muscle relaxation
	**Writing exercise**	Fine motor exercise
	**Physiotherapy**	Hand stretches
**16**	**Collecting positive experiences**	A short video about collecting positive experiences: Exploring why we tend to focus on negative events and how we can shift our attention to positive ones.
	**Activity choices**	Guided imagery to a safe place/body scan/breathing awareness practice
	**Coordination**	Movement sequences with rhythm
	**Physiotherapy**	Motor skills practice 3
**17**	**Activity choices**	Body scan/box breathing/progressive muscle relaxation practice
	**Flexibility and adaptability**	Video
	**Physiotherapy**	Neck stretches
**18**	**Worry cycle**	A video explaining the psychosomatic cycle: how extreme interpretations of unpleasant feelings can intensify stress and anxiety, along with techniques to break free from this cycle.
	**Activity choices**	Progressive muscle relaxation/breathing awareness /guided imagery to a safe place
	**Toilet paper folding practice**	Video
	**Physiotherapy**	Opening and closing the palms
**19**	**Avoidance and struggle**	A short video on avoidance and struggle, explains that while avoiding or struggling with unpleasant feelings may provide short-term relief, it is ultimately ineffective in the long term.
	**Activity choices**	Guided imagery for medication intake/box breathing/body scan
	**Fine motor skills**	Practicing with a pen
	**Neuro-dance**	wide and open movements
**20**	Acceptance	A short video on acceptance: an essential aspect of emotional regulation and coping with difficult situations is the ability to accept the current reality as it is. This approach helps us navigate challenges without surrendering to them.
	**Activity choices**	Body scan/breathing awareness/guided imagery practice
	**Coordination**	Finger movement sequences
	**Physiotherapy**	Getting up from a chair
**21**	**Practicing fine motor skills**	Ball exercise
	**Program summary**	

3. Study Outcomes

Primary Outcome
MDS-UPDRS total scores (sum of parts I, II & III): The inter-group difference in the mean change from baseline (Visit 1) to the end of the study period (Visit 2) in the sum of MDS-UPDRS score for sum of parts I, II & III.

**MDS-UPDRS Assessments**[1]	
**Parts** I-IV	MDS-UPDRS score sum of parts I, II, III & IV: the inter-group difference in the mean change from baseline (Visit 1) to the end of the study period (Visit 2) in MDS-UPDRS score for sum of parts I, II, III & IV.
**Individual parts**	MDS-UPDRS score for each of parts I, II, III & IV individually: the inter-group difference in the mean change from baseline (Visit 1) to the end of the study period (Visit2) in MDS-UPDRS score in each part (I-IV) separately.
**Parts** II & III	MDS-UPDRS score sum of parts II & III: The inter-group difference in the mean change from baseline (Visit 1) to the end of the study period (Visit 2) in MDS-UPDRS score for sum of parts II & III.
**Parts** I & II	MDS-UPDRS score sum of parts I & II: The inter-group difference in the mean change from baseline (Visit 1) to the end of the study period (Visit 2) in MDS-UPDRS score for sum of parts I & II.
**Speech**	MDS-UPDRS sections related to speech and oral muscle function total = MDS-UPDRS Speech (#2.1; #2.2; #2.3; #2.4; #3.1; #3.2): The inter-group difference in the mean change from baseline (Visit 1) to the end of the study period (Visit 2) in the MDS-UPDRS Speech sections listed above.
**Fine hand motor**	MDS-UPDRS related to fine motor skills and hand movement total = MDS-UPDRS fine hand motor (#2.4; #2.5, #2.6, #2.7; #2.8, #3.4; #3.5, #3.6 #3.15, #3.16). The inter-group difference in the mean change from baseline (Visit 1) to the end of the study period (Visit 2) in the MDS-UPDRS fine hand motor sections listed above.
**Proportion of Responders by MDS-UPDRS Improvement**	The inter-group difference in the proportion of subjects in each group, achieving predefined levels of change in the MDS-UPDRS total score from baseline (Visit 1) to the end of the study period (Visit 2). Specifically, we assessed the proportion of subjects reaching at least the following thresholds:<br>o A reduction of at least 5.5 points ("Minimal Detectable Response Rate")<br>o A reduction of at least 6.7 points ("Minimal (MCID) Response Rate")<br>o A reduction of at least 10 points<br>o A reduction of at least 20% in the score compared to baseline visit score<br>o A reduction of at least 30% in the score compared to baseline visit score

**PD-related Outcomes**	
**PDQ-39**[2]	The inter-group difference in the mean change from baseline (Visit 1) to the end of the study period (Visit 2) in PDQ-39. A summary index is then calculated as the sum of the total score of the dimensions divided by the number of dimensions, i.e. sum of dimension scores /8. If any item score is missing, the relevant dimension score and the summary index will be missing.
**VHI-HEB**[3]	The inter-group difference in the mean change from baseline (Visit 1) to the end of the study period (Visit 2) in VHI total score and in each subscale (functional/physical/emotional) separately.
**PDSS-2**[4]	The inter-group difference in the mean change from baseline to the end of the study period (Visit 2) in PDSS score.
**Psychological & Cognitive Outcomes**	
**TMT A&B**[5]	The inter-group difference in the mean change from baseline (Visit 1) to the end of the study period (Visit 2) in TMT A & B total score and in each of them separately.
**BDI-II**[6]	The inter-group difference in the mean change from baseline (Visit 1) to the end of the study period (Visit 2) in BDI-II total score.
**STAIT-5/ STAIS-5**[7]	The inter-group difference in the mean change from baseline (Visit 1) to the end of the study period (Visit 2) in STAIT-5/STAIS-5 total score.
**BRCS**[8]	The inter-group difference in the mean change from baseline (Visit 1) to the end of the study period (Visit 2) in BRCS total score.
**PSS-10**[9]	The inter-group difference in the mean change from baseline (Visit 1) to the end of the study period (Visit 2) in PSS-10 total score.
**NHC-SF**[10]	The inter-group difference in the mean change from baseline (Visit 1) to the end of the study period (Visit 2) in MHC-SF total score
**Brain Imaging Outcomes**	
**Resting state fmri**	Inter-group resting state fMRI connectivity changes from baseline (Visit 1) to the end of the study period (Visit 2) (peak connectivity coordinates, significance value, and cluster size). Contrast: DopApp arm vs. placebo arm.
**DopApp use Outcomes**	
App Engagement	Measuring app usage and activity levels. App engagement will be calculated for the trial group as number of days the users completed their daily digital treatment protocol during trial period.
**Safety Outcomes**	
Adverse events	Adverse events.

4. Supplementary Tables

Supplementary Table 1: Mean daily engagement time with DopApp

Engagement Context	**Total App Usage** (min/day)	**Games Usage** (min/day)	**Media Usage** (min/day)
**All Time**	47.30±11.69	29.01±9.99	18.29±3.64
**Daily protocol use**	36.66±4.16	22.68±3.67	13.98±1.19
**Self-initiated use**	17.11±11.57	14.91±11.43	8.58±4.83

Supplementary Table 2: Max head motion

	PRE		POST			
	**Translation**	**Rotation (Deg)**	**Translation**	**Rotation (Deg)**	**Time t-test**	
**DopApp**	0.99±0.54	1.32±0.68	0.97±0.55	1.15±0.77	0.852	0.357
**Placebo**	0.80±0.52	1.04±0.65	0.87±0.51	1.12±0.87	0.597	0.709
**Group t-test**	0.159	0.105	0.491	0.850		

Supplementary Table 3: Response rate analysis across multiple clinical thresholds

**Threshold**	**DopApp %**	**Placebo %**	***P*-value**
≤ -5.5 points	75.0%	15.8%	0.0003
≤ -6.7 points (MCID)	65.0%	15.8%	0.0031
≤ -10 points	45.0%	5.3%	0.0084
≥ 20% reduction	65.0%	15.8%	0.0031
≥ 30% reduction	50.0%	0.0%	0.0004

*Response rates in the DopApp™ and placebo groups across predefined clinical improvement thresholds in total MDS-UPDRS scores. Response rates were calculated for each group, and between-group differences were tested using Fisher's exact test.

Supplementary Table 4: Primary outcome MDS-UPDRS total score sensitivity analyses

Analysis type	Comparison		Effect size
**Outliers' exclusion** (n=19 DopApp; n=17 control)	DopApp, mean±SD<br>Control, mean ±SD<br>Treatment difference, mean [t(df), p value]	-8.74 ± 5.71<br>-3.35 ± 3.72<br>5.38 [3.3 (34), *P* = 0.002]	1.11
**Including all mITT** group (n=21 DopApp; n=21 control)	DopApp, mean±SD<br>Control, mean±SD<br>Treatment difference, mean (t(df), p value)	-9.24 ± 7.18<br>-2.19 ± 7.47<br>7.05 [3.12 (40) *P* = 0.003]	0.96
**Non-parametric** statistical tests using the Wilcoxon rank test	DopApp, median Visit 1/Visit 2<br>Control, median Visit 1/Visit 2<br>Median difference between visits DopApp vs control<br>P value	34.5/24.0<br>39.0/34.0<br>-9.0 vs. -3.0<br>*P* = 0.0003	
**Linear regression with covariants**	Inter-Group<br>MDS-UPDRS total score at baseline<br>LEDD at Baseline<br>Time (years) since PD diagnosis	*P* = 0.0003<br>*P* = 0.91<br>*P* = 0.18<br>*P* = 0.1	

Supplementary Table 5: Change from baseline in exploratory outcomes

Variable name	Baseline DopApp Mean ± SD (n=20)	Change from baseline DopApp Mean ± SD (n=20)	Baseline Control Mean ± SD (n=19)	Change from baseline Control Mean ± SD (n=19)	Treatment difference Mean, t(df) *P*-value	Effect size Cohens d
**MDS-UPDRS assessments**						
**Parts** I, II & III	36.55±12.49	-9.7 ± 7.03	42.42±18.58	-1.95 ± 5.57	7.75, 3.8(37), p=0.0005	1.22
**Parts** I-IV	39.95±13.5	-10.15 ± 7.5	47.11±20.1	-1.79 ± 6.8	8.36, 3.64(37), p=0.0008	1.17
**Part** I	5.5±4.36	-0.6 ± 3.39	8.16±5.09	-0.79 ± 3.07	-0.19, -0.18(37), p=0.8561	-0.06
**Part II**	6.85±3.13	-1.75 ± 2.49	8.05±7.3	-0.26 ± 1.79	1.49, 2.13(37), p=0.0398	0.68
**Part III**	24.2±9.17	-7.35 ± 6.29	26.21±13.36	-0.89 ± 4.68	6.46, 3.62(37), p=0.0009	1.16
**Part IV**	3.4±3.52	-0.45 ± 2.56	4.68±3.65	0.16 ± 2.99	0.61, 0.68(37), p=0.4988	0.22
**Parts** II & III	31.05±10.85	-9.1 ± 6.2	34.26±16.9	-1.16 ± 4.5	7.94, 4.56(37), p=0.0001	1.46
**Parts** I & II	12.35±6.35	-2.35 ± 5.3	16.21±10.57	-1.05 ± 3.87	1.3, 0.87(37), p=0.3904	0.28
**Speech**[a]	3.65±2.41	-0.45 ± 2.01	3.95±2.22	0.16 ± 2.29	0.61, 0.88(37), p=0.3838	0.28
**Fine hand motor**[b]	10.35±3.84	-3.1 ± 3.92	11.95±6.62	-0.21 ± 3.17	2.89, 37(37), p=0.0161	0.81
**Questionnaires**						
**PDQ-39**	19.84±12.29	-6.46 ± 10.67	24.7±15.21	-3.42 ± 4.31	3.03, 1.15(37), p=0.256	0.37
**VHI-HEB**	16.1±12.24	-0.55 ± 12.08	21.74±25.09	0.47 ± 7.27	1.02, 0.32(37), p=0.752	0.1
**PDSS-2**	14.75±8.96	-2.7 ± 6.09	22.42±12.5	-4.21 ± 11.5	-1.51, -0.52(37), p=0.609	-0.17
**TMT-A**	48.25±17.03	-9.6 ± 10.46	50.63±17.42	-5.47 ± 10.6	4.13, 1.22(37), p=0.229	0.39
**TMT A+B**	145.35±40.11	-23.4 ± 31.46	182.42±75.11	-21.42 ± 32.94	1.98, 0.19(37), p=0.849	0.06
**BDI-II**	8.9±7.14	-3.5 ± 5.02	9.16±6.59	0.05 ± 4.31	3.55, 2.36(37), p=0.023	0.76
**STAIT-5**	6.85±2.25	-0.9 ± 1.55	7.68±2.65	-0.53 ± 1.78	0.37, 0.7(37), p=0.488	0.22
**STAIS-5**	8.1±2.34	-1.1 ± 2.1	8.74±3.31	-0.47 ± 1.71	0.63, 1.02(37), p=0.315	0.33
**BRCS**	16.5±3	0.25 ± 2.47	14.58±4.39	2.11 ± 4.91	1.86, 1.5(37), p=0.141	0.48

**PSS-10**	12.05±5.59	-3.35 ± 4.46	14.42±5.55	-1.95 ± 3.47	1.4, 1.09(37), p=0.282	0.35
**MHC-SF**	58.5±13.22	4.5 ± 9.22	58.16±11.03	2.11 ± 9.88	-2.39, -0.78(37), p=0.439	-0.25

[a]MDS-UPDRS Speech (#2.1; #2.2; #2.3; #2.4; #3.1; #3.2), [b]MDS-UPDRS fine hand motor (#2.4; #2.5, #2.6, #2.7; #2.8, #3.4; #3.5, #3.6 #3.15, #3.16). Abbreviations: PDSS-2, Parkinson's Disease Sleep Scale; PDQ-39, PD Questionnaire 39-item; VHI, Voice Handicap Index; PSS-10, Perceived Stress Scale; STAIT-5/STAIS-5, Brief State and Trait Anxiety Inventory; BDI-II, Beck's Depression Inventory II; MHC-SF, Mental Health Continuum Short Form; BRCS, Brief Resilient Coping Scale; TMT, Trail Making Test.

Supplementary Table 6: Longitudinal changes in connectivity measures: Motor circuit

Seed-HCPex ID*	Seed-HCPex area	Cluster (x,y,z)	k	T(29)	*P*-FWE	*P*-FDR	*P*-UNC	Brain area	BA	Cluster HCPex ID*
413	VLp	+48 -8 +48	177	4.42	< 0.001	< 0.001	< 0.001	M1	4	211
		-24 -22 60	100	4.01	< 0.001	< 0.001	< 0.001	M1	4	31
379	VLa	+46 -12 +50	97	3.50	0.350	0.142	< 0.001	M1	4	211
380	VLa	NS								
412	VLa	NS								

* HCPex atlas,[11] FWE, familywise error, FDR, false discovery rate, UNC, uncorrected, X, Y, Z MNI coordinates, BA, Brodmann area, VLa, ventral lateral anterior, VLp, ventral lateral posterior; M1, primary motor cortex, post>pre-intervention; DopApp > Placebo, *P*-value was analyzed at the **peak-level** within VL-M1 mask, k, cluster size, NS, not significant. Data supporting the results shown in main **Figure 3**

Supplementary Table 7: Longitudinal changes in connectivity measures: Limbic circuit

Seed-HCPex ID*	Seed-HCPex area	Peak Cluster (x,y,z)	k	T(29)	*P*-FWE	*P*-FDR	*P*-UNC	Brain area	BA	Cluster HCPex ID*
		+56, +04, -36	650	4.45	<0.001	<0.001	<0.001	TG	20	274
		+58, -10, -8	203					Auditory5	22	241
		+56, -02, -24	196					STS	21	245
		+52, +06, -34	53					TG	38	274
		+62, -14, +6	51					Auditory4	41	240
		+62, -10, -18	50					TE	21	269
361	AV	+50, -26, +52	555	4.25	<0.001	<0.001	<0.001	PSC	1	208
		-54, -66, +16	346	4.19	0.004	0.001	<0.001	LOC	39	99
		+28, -20, -24	313	6.67	0.008	0.002	<0.001	ParaH	36	262
		+26, -16, -20	89	4.36				Hippocampus		260
		+10, +54, +24	255	4.61	0.027	0.005	<0.001	dlPFC	9	319
		-12, -52, +18	237	4.15	0.039	0.007	<0.001	PCC	23	129
		+02, +60, -10	230	4.34	0.046	0.007	<0.001	mPFC	10	315
		-22, -12, -24	215	3.46	0.063	0.008	<0.001	Hippocampus		80
394	AV	+02, +58, -20	202	3.87	0.087	0.056	0.001	mPFC	10	315
369	MDl	NS								
402	MDl	-32, -74, +14	409	5.12	0.001	<0.001	<0.001	LOC	19	25
		-40, +02, +42	210	4.14	0.062	0.026	<0.001	PreMot	6	47
370	MDm	-38, -68, +08	305	4.59	0.010	0.005	<0.001	MT(V5)	19	23
		-44, -68, +8	132					LOC	19	18
403	MDm	-42, +00, +38	224	4.21	0.058	0.039	<0.001	PreMot	6	47
387	Amyg	NS								
420	Amyg	NS								

* HCPex atlas,[11] FWE, familywise error, FDR, false discovery rate, UNC, uncorrected, X, Y, Z MNI coordinates, BA, Brodmann area, AV, anteroventral , MDl, mediodorsolateral parvocellular, MDm, mediodorsomedial magnocellular limbic-related nuclei of the thalamus; TG, temporal gyrus, STS, superior temporal sulcus, PSC, primary sensory cortex, TE, inferior temporal cortex, LOC, lateral occipital cortex, ParaH, parahippocampal gyrus, dlPFC, dorsolateral prefrontal cortex, mPFC, medial prefrontal cortex, PreMot, premotor cortex, MT, medial temporal, whole-brain, Post>Pre-intervention; DopApp > Placebo, k, cluster size, NS, not significant. Data supporting the results shown in main **Figure 4.**

Supplementary Table 8: Significant associations between alterations in brain connectivity and changes in non-imaging data

Non-imaging Parameter	Seed ID*	Target Area	BA	Peak Cluster (x,y,z)	r	*P*-value	r	*P* -value
**UPDRS/ Questionnaire Parameters**					**DopApp**		**Placebo**	
MDS-UPDRS Part III	413	M1	4	+36 -26 +60	-0.72	0.0012	0.13	0.650
MDS-UPDRS Part III	413	M1	4	-28 -22 +56	-0.64	0.0053	0.39	0.174
MDS-UPDRS Part II	369	dlPFC	9	+40 +24 +20	-0.79	0.0002	0.31	0.281
MDS-UPDRS Part II	370	PSC	1	+48 -16 +14	-0.86	<0.0001	0.30	0.304
MDS-UPDRS Part II	370	INS	13	+38 -16 +12	-0.65	0.0049	0.24	0.415
MDS-UPDRS Part II	370	PSC	1	+54 -18 +50	-0.79	0.0002	-0.12	0.694
MDS-UPDRS Part II	402	SMC	6	-2 -14 +50	-0.84	<0.0001	-0.37	0.194
MDS-UPDRS Part II	402	SMC	6	+2 +6 +62	-0.86	<0.0001	-0.04	0.894
MDS-UPDRS Part II	402	SecVis	18	+12 -76 +26	-0.79	0.0002	-0.06	0.842
MDS-UPDRS Part II	402	SMG	40	+62 -22 +26	-0.82	<0.0001	-0.04	0.885
MDS-UPDRS Part II	403	Broca45	45	-28 +28 +16	-0.85	<0.0001	-0.15	0.616
MDS-UPDRS Part II	403	INS	13	-32 +20 +12	-0.68	0.0028	-0.20	0.502
MDS-UPDRS Part II	403	dlPFC	46	+46 +34 +18	-0.78	0.0002	-0.01	0.960
MDS-UPDRS Part II	420	AV		+6 -4 +12	-0.66	0.0043	-0.47	0.087
MDS-UPDRS Part II	420	CD		+10 +8 +6	-0.79	0.0002	0.06	0.835
BDI-II	361	HIPP		-18 -12 -16	-0.65	0.0046	-0.06	0.843
BDI-II	361	AMY		-16 -14 -18	-0.63	0.0070	0.17	0.558
BDI-II	387	INS	13	+38 +2 +6	-0.66	0.0041	0.43	0.124
BDI-II	387	INS	13	+56 -14 +8	-0.77	0.0003	-0.10	0.724
BDI-II	420	AV		+8 -10 +14	-0.75	0.0006	-0.16	0.583
BDI-II	420	CD		+10 +6 +10	-0.75	0.0005	-0.09	0.748
BDI-II	420	CD		-10 +10 +8	-0.71	0.0014	-0.29	0.311
BDI-II	420	PreMot	6	-50 -10 +4	-0.86	<0.0001	-0.29	0.312
BDI-II	420	M1	4	-46 -12 +8	-0.68	0.0024	-0.48	0.086
**App Engagement Parameters**								
Games: Daily avg (min/day)	380	PreMot	6	-46 +8 +32	0.75	0.0005		
Games: Daily avg (min/day)	402	SMC	6	-16 -8 +68	0.78	0.0002		
Games: Daily avg (min/day)	403	SMC	6	+12 -6 +76	0.83	<0.0001		
Games: Total time (min)	403	SMC	6	+8 -8 +74	0.84	<0.0001		
Games: Total time (min)	403	SMC	6	-14 -4 +76	0.78	0.0002		
Media: Daily avg (min/day)	361	OFC	11	-20 +12 -16	0.80	0.0001		
Media: Daily avg (min/day)	403	dlPFC	9	+48 +30 +25	0.85	<0.0001		
Media: Daily avg (min/day)	403	aPFC	10	-38 +56 +6	0.75	0.0005		
Media: Total time (min)	387	CD		+10 +6 +10	0.72	0.0012		
Media: Total time (min)	387	PreMot	6	-36 +2 +58	0.85	<0.0001		
Media: Total time (min)	403	dlPFC	9	+48 +30 +24	0.84	<0.0001		
Media: Total time (min)	403	aPFC	10	-38 +54 +6	0.76	0.0004		
Sensorimotor: Daily avg (min/day)	380	PreMot	6	-46 +4 +30	0.79	0.0002		
Sensorimotor: Daily avg (min/day)	402	SMC	6	-16 -8 +68	0.83	<0.0001		
Sensorimotor: Daily avg (min/day)	403	SMC	6	+12 -4 +76	0.80	0.0001		
Sensorimotor: Daily avg (min/day)	403	SMC	6	-14 -4 +78	0.77	0.0003		
Sensorimotor: Daily avg (min/day)	403	InfTG	20	+30 -8 -42	0.88	<0.0001		
Sensorimotor: Daily avg (min/day)	413	M1	4	-24 -34 72	0.81	<0.0001		
Sensorimotor: Daily avg (min/day)	413	SMC	6	-10 0 72	0.82	<0.0001		

Sensorimotor: Daily avg (min/day)	413	Fusiform	37	-62 -66 +2	0.80	0.0001
Sensorimotor: Daily avg (min/day)	413	M1	4	+22 -28 +76	0.75	0.0005
Sensorimotor: Total time (min)	403	SMC	6	+16 -6 +78	0.79	0.0002
Sensorimotor: Total time (min)	403	SMC	6	-18 -2 +76	0.85	<0.0001
Sensorimotor: Total time (min)	403	InfTG	20	+30 -8 -42	0.83	<0.0001
Sensorimotor: Total time (min)	413	SMC	6	-12 0 +76	0.84	<0.0001
Sensorimotor: Total time (min)	413	SMC	6	+8 -18 +72	0.68	0.0028
Extra Games: Daily avg (min/day)	380	Broca44	44	-62 +8 +26	0.81	<0.0001
Extra Games: Daily avg (min/day)	403	SMC	6	-14 -4 +78	0.79	0.0001
Extra Games: Daily avg (min/day)	413	PreMot	6	-46 +4 +36	0.80	0.0001
Extra Games: Daily avg (min/day)	413	SMC	6	-30 -6 +58	0.77	0.0003
Extra Games: Daily avg (min/day)	413	AG	39	-58 -56 +12	0.76	0.0004
Extra Games: Daily avg (min/day)	420	OFC	11	-19 +63 -14	0.70	0.0018
Extra Games: Daily avg (min/day)	420	aPFC	10	-34 +56 -6	0.85	<0.0001
Extra Games: Total time (min)	420	aPFC	10	+42 +58 -14	0.77	0.0003
Extra Media: (N of days)	403	dlPFC	9	+48 +32 +22	0.82	<0.0001
Extra Media: Daily avg (min/day)	361	mPFC	10	-12 +65 +21	0.82	<0.0001
Extra Media: Daily avg (min/day)	361	mPFC	10	-24 +52 +32	0.62	0.0080
Extra Media: Daily avg (min/day)	403	aPFC	10	-38 +52 +10	0.89	<0.0001
Extra Media: Daily avg (min/day)	403	dlPFC	9	+30 +38 +20	0.83	<0.0001
Extra Media: Total time (min)	403	dlPFC	46	-50 +30 +14	0.77	0.0003
Extra Media: Total time (min)	403	aPFC	10	-38 +54 +6	0.86	<0.0001
Extra Media: Total time (min)	403	dlPFC	9	+50 +30 +24	0.82	<0.0001
Extra Sensorimotor: (N of days)	413	SMC	6	-12 +2 +76	0.77	0.0003
Extra Sensorimotor: Daily avg (min/day)	379	SMC	6	-20 -6 +60	0.82	<0.0001
Extra Sensorimotor: Daily avg (min/day)	380	PreMot	6	-40 -12 +60	0.80	0.0001
Extra Sensorimotor: Daily avg (min/day)	380	PreMot	6	-50 0 +34	0.76	0.0005
Extra Sensorimotor: Daily avg (min/day)	413	SMC	6	-20 +2 +54	0.86	<0.0001
Extra Sensorimotor: Daily avg (min/day)	413	AG	39	-56 -56 +10	0.79	0.0002
Extra Sensorimotor: Total time (min)	380	Broca44	44	-48 +6 +26	0.71	0.0014
Extra Sensorimotor: Total time (min)	413	Fusiform	37	-62 -64 +2	0.79	0.0001
Extra Sensorimotor: Total time (min)	413	SensAssoc	5	-26 -36 +70	0.80	0.0001

* HCPex atlas,[11] X, Y, Z MNI coordinates, BA, Brodmann area, AV, anteroventral , MDl, mediodorsolateral parvocellular, MDm, mediodorsomedial magnocellular limbic-related nuclei of the thalamus; CD, caudate, AMY, amygdala, INS, insula, HIPP, hippocampus, M1, primary motor cortex, aPFC, anterior prefrontal cortex, dlPFC, dorsolateral prefrontal cortex, mPFC, medial prefrontal cortex, PreMot, premotor cortex, SMC, supplementary motor complex, OFC, orbitofrontal cortex, ParsOrb, pars orbitalis, TG, temporal Gyrus, PSC, primary sensory cortex, SMG, suppramarginal gyrus, AG, angular gyrus; r,p Pearson's correlation coefficient. Data supporting the results shown in main **Figures 3**-**5**.

5. Supplementary Figures

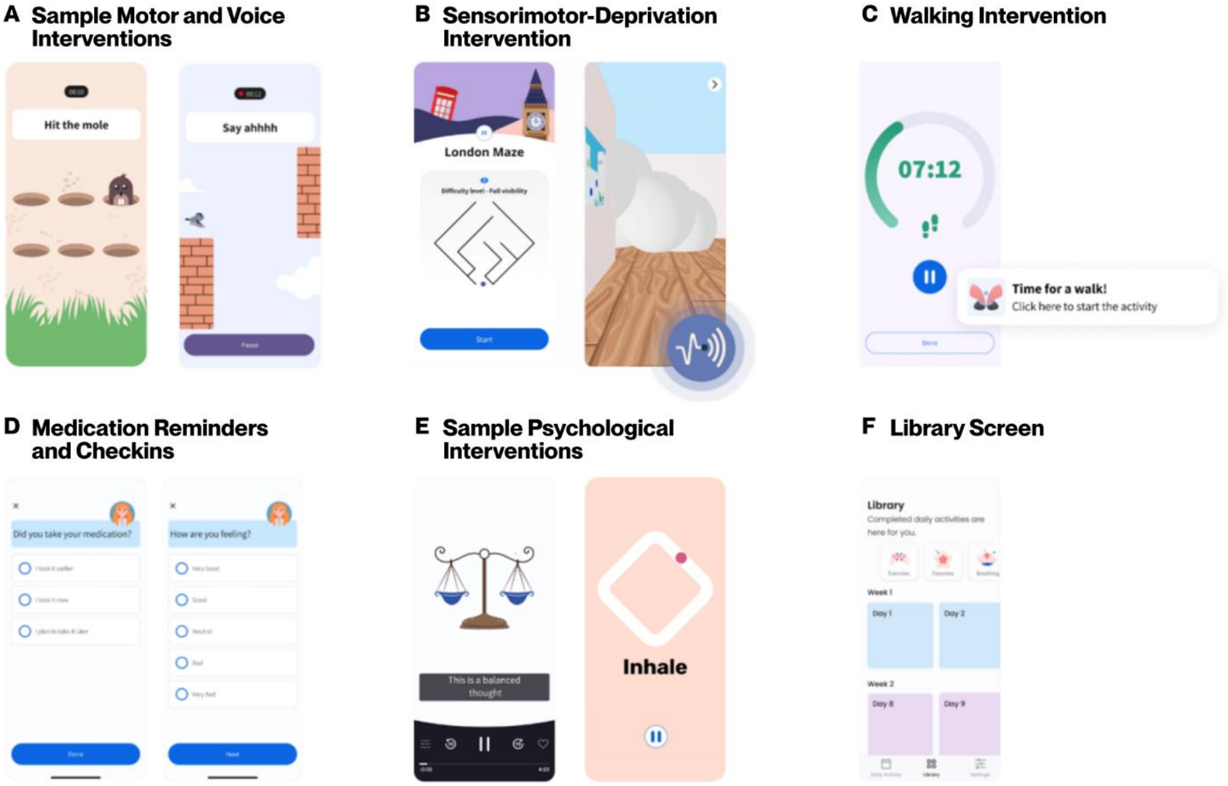

Supplementary Figure 1: DopApp™ Sample Screens. a. Sample motor and voice interventions: fine motor game of hit-the-mole (left) and pitch modulation training game (right). **b.** Sample sensorimotor-deprivation spatial memory screens: The exercise features virtual spatial navigation exercises that utilize digital 3D Hebb-Williams mazes and incorporate both allocentric and egocentric navigation techniques. This exercise is implemented through a progressive, three-step vision-deprivation sensorimotor training process, designed to increase navigation complexity over time. Each maze trial starts with a top-down 2D map view for allocentric navigation (left). Participants then navigate the maze fully sighted, using auditory cues for spatial information, such as distance from walls. After completing this phase, the 2D map is shown again, followed by a more challenging 3D navigation phase where 50% of the maze is masked (right). In the final phase, participants navigate blindfolded using only auditory feedback, employing a sensory substitution strategy. This approach aims to enhance brain connectivity, spatial learning, and balance across sensory and cognitive networks,[12] **c.** Walking intervention: reminder (right) and walking timer. **d.** medication reminder (left) and check-in (right). **e.** Sample psychological interventions: CBT module on balanced thoughts (left) and breathing exercise (right). **f.** Library screen. A detailed explanation of the intervention protocol is provided in the Supplementary Information.

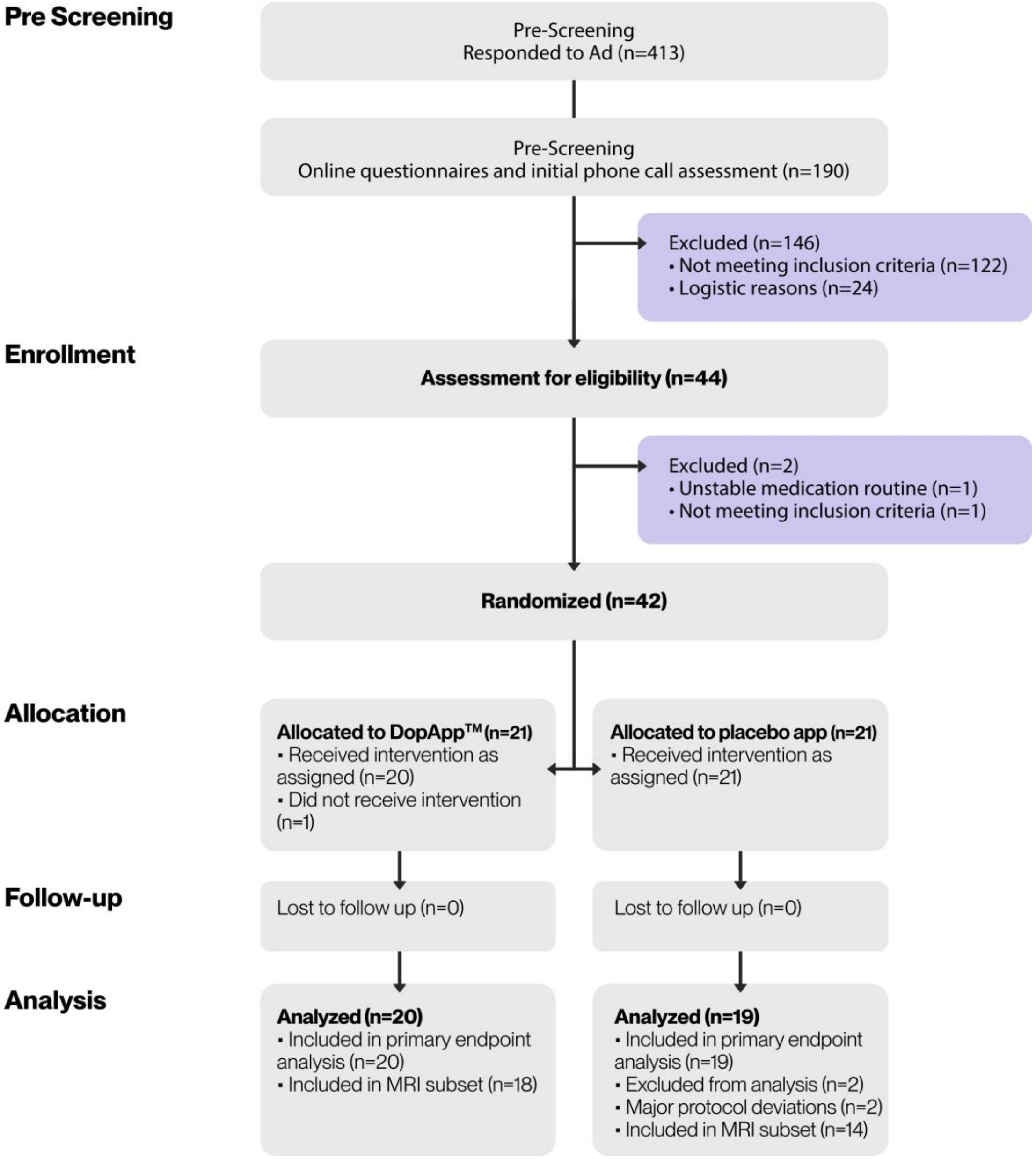

Supplementary Figure 2: Study flowchart. A total of 413 individuals responded to the study advertisement, of whom 190 completed the prescreening questionnaire. Many were excluded for not meeting eligibility criteria or due to technical issues. Ultimately, 44 PwP were screened, 42 met the inclusion criteria and were enrolled and randomized. One participant in the DopApp™ group withdrew consent prematurely, and two in the placebo group were excluded due to major protocol deviations. A total of 39 participants completed the study per protocol.

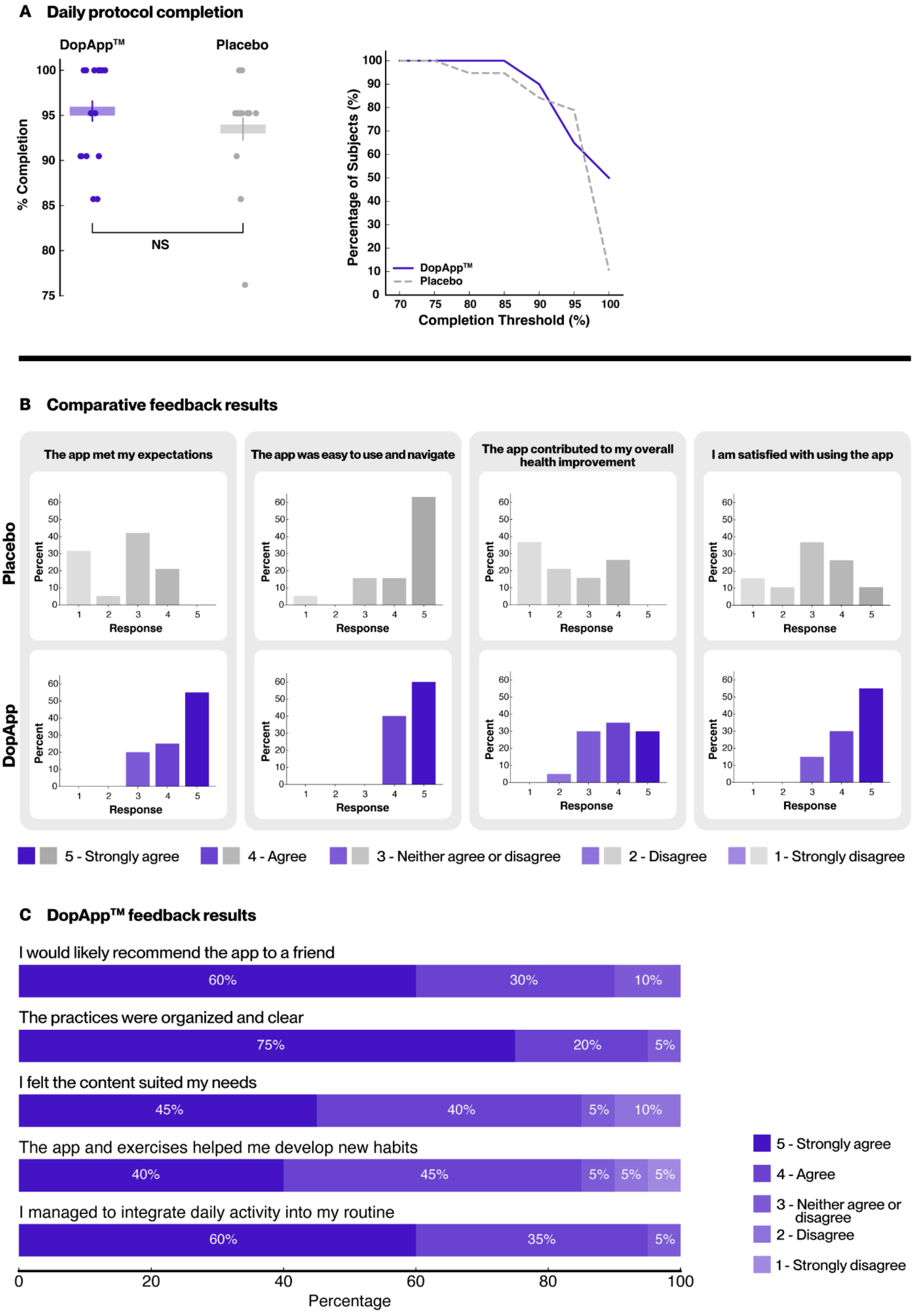
A Daily protocol completion
DopApp™
Placebo
% Completion
NS
Percentage of Subjects (%)
Completion Threshold (%)
DopApp™
Placebo
B Comparative feedback results
The app met my expectations
The app was easy to use and navigate
The app contributed to my overall health improvement
I am satisfied with using the app
Placebo
DopApp
Percent
Response
5 - Strongly agree
4 - Agree
3 - Neither agree or disagree
2 - Disagree
1 - Strongly disagree
C DopApp™ feedback results
I would likely recommend the app to a friend
60%
30%
10%
The practices were organized and clear
75%
20%
5%
I felt the content suited my needs
45%
40%
5%
10%
The app and exercises helped me develop new habits
40%
45%
5%
5%
5%
I managed to integrate daily activity into my routine
60%
35%
5%
Percentage
5 - Strongly agree
4 - Agree
3 - Neither agree or disagree
2 - Disagree
1 - Strongly disagree

Supplementary Figure 3: Adherence and satisfaction survey results for the DopApp™ and placebo interventions. a. Daily protocol completion rates between DopApp™ and placebo groups across the three-week intervention, with both groups demonstrating high compliance and no significant between-group differences (t(37)=1.15, *P*=0.26, DopApp™, n=20; placebo, n=19). Left, individual and group-level compliance percentages; Horizontal lines represent the group means, and vertical error bars indicate the SEM. NS = not significant. Right, cumulative percentage of participants meeting increasing daily completion thresholds. **b.** Comparative feedback ratings between placebo and DopApp™ participants across four domains: expectation matching, ease of use, perceived health improvement, and overall satisfaction. Item scale: 5 (strongly agree), 4 (agree), 3 (neither agree or disagree), 2, (disagree) and 1 (strongly disagree) **c.** Detailed feedback from DopApp™ users on five specific statements related to app usability, content clarity, habit formation, and daily integration. Bar graphs indicate the percentage of participants selecting each response level (1–5), with darker shades indicating stronger agreement.

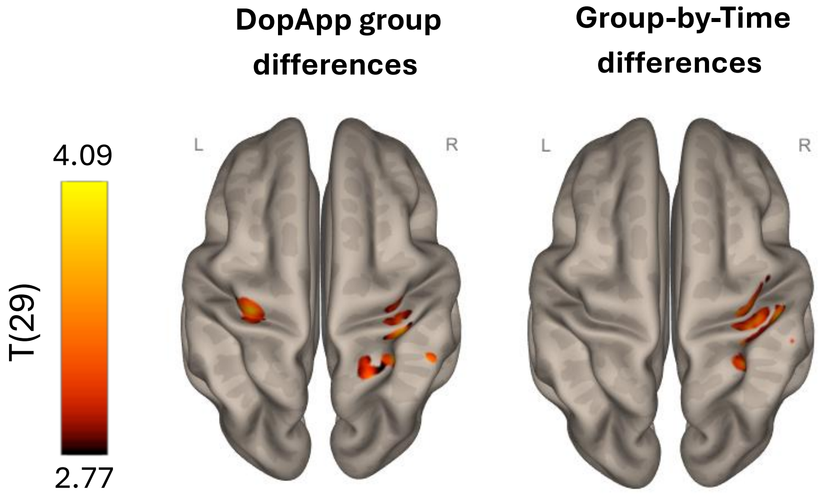

Supplementary Figure 4: Thalamomotor circuit connectivity analysis. Whole-brain seed-to-voxel connectivity maps of longitudinal differences through the thalamomotor circuit. (Seed: right VLp) right: group-by-time interaction model of the resting state whole-brain imaging data, DopApp™, n=17; placebo, n=14. Left: DopApp™ group POST > PRE-intervention analysis, DopApp™, n=17. Statistical analysis: voxel level, $P < 0.005$, cluster level, $P < 0.05$, FDR corrected, parametric stats. Brain images were generated using CONN (RRID:SCR_009550) https://web.conn-toolbox.org, v22a.

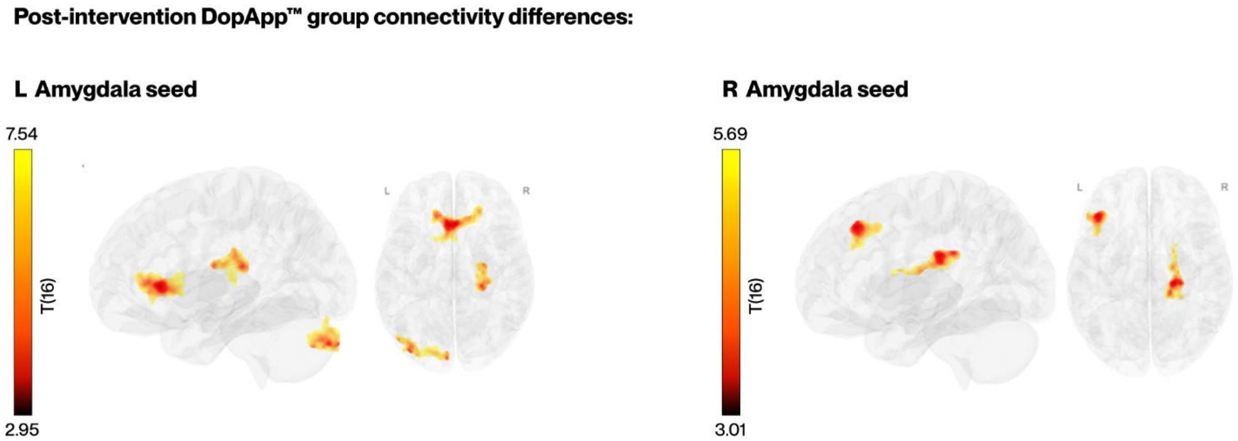

Supplementary Figure 5: Post-intervention alterations in the DopAppTM group. Seed-to-voxel maps of longitudinal test group differences in the right and left amygdala seeds. Within the limbic network, increased post intervention connectivity was demonstrated between the left amygdala and the left caudate (peak:+2, +26, +2; T=7.54; k=543; *P* < 0.001 FDR corrected, N=17), and between the right amygdala and the right thalamus (peak:+18, -14, +14; T=3.94; k=61; *P* < 0.05 FDR corrected, n=17) Statistical analysis: voxel level, *P* < 0.005, cluster level, *P* < 0.05, FDR corrected, parametric stats. Brain images were generated using CONN (RRID:SCR_009550) https://web.conn-toolbox.org, v22a.

Supplementary references

1 Goetz, C. G. *et al.* Movement Disorder Society-sponsored revision of the Unified Parkinson's Disease Rating Scale (MDS-UPDRS): scale presentation and clinimetric testing results. *Mov Disord* **23**, 2129-2170, doi:10.1002/mds.22340 (2008).

2 Jenkinson, C., Fitzpatrick, R., Peto, V., Greenhall, R. & Hyman, N. The Parkinson's Disease Questionnaire (PDQ-39): development and validation of a Parkinson's disease summary index score. *Age and ageing* **26**, 353-357 (1997).

3 Jacobson, B. H. *et al.* The voice handicap index (VHI) development and validation. *American journal of speech-language pathology* **6**, 66-70 (1997).

4 Trenkwalder, C. *et al.* Parkinson's disease sleep scale—validation of the revised version PDSS-2. *Movement Disorders* **26**, 644-652 (2011).

5 Tombaugh, T. N. Trail Making Test A and B: normative data stratified by age and education. *Archives of clinical neuropsychology* **19**, 203-214 (2004).

6 Beck, A. T., Steer, R. A. & Brown, G. Beck depression inventory–II. *Psychological assessment* (1996).

7 Zsido, A. N., Teleki, S. A., Csokasi, K., Rozsa, S. & Bandi, S. A. Development of the short version of the spielberger state—trait anxiety inventory. *Psychiatry research* **291**, 113223 (2020).

8 Sinclair, V. G. & Wallston, K. A. The development and psychometric evaluation of the Brief Resilient Coping Scale. *Assessment* **11**, 94-101 (2004).

9 Cohen, S., Kamarck, T., Mermelstein, R. J. J. o. h. & behavior, s. A global measure of perceived stress. 385-396 (1983).

10 Lamers, S. M., Westerhof, G. J., Bohlmeijer, E. T., ten Klooster, P. M. & Keyes, C. L. J. J. o. c. p. Evaluating the psychometric properties of the mental health continuum-short form (MHC-SF). **67**, 99-110 (2011).

11 Huang, C.-C., Rolls, E. T., Feng, J. & Lin, C.-P. An extended Human Connectome Project multimodal parcellation atlas of the human cortex and subcortical areas. *Brain Structure and Function* **227**, 763-778 (2022).

12 Amedi, A., Shelly, S., Saporta, N. & Catalogna, M. Perceptual learning and neural correlates of virtual navigation in subjective cognitive decline: A pilot study. *iScience* (2024).
